# Simulating neutron protein crystallography experiments: applications to the development of the NMX instrument at ESS

**DOI:** 10.1107/S205979832600584X

**Published:** 2026-06-21

**Authors:** Mads Bertelsen, Peter K. Willendrup, Sunyoung Yoo, Adriano Meligrana, David McDonagh, Justin Bergmann, Esko Oksanen, Aaron D. Finke

**Affiliations:** a Data Management and Scientific Computing, European Spallation Source ERIC, Kongens Lyngby, Denmark; b European Spallation Source ERIC Lund Sweden; c Department of Computer, Control, and Management Engineering, Sapienza University of Rome, Rome, Italy; d STFC Rutherford Appleton Laboratory Didcot United Kingdom; e LINXS – Institute of Advanced Neutron and X-ray Science, Lund, Sweden; f Division of Computational Chemistry, Lund University, Lund, Sweden; University of Manchester, United Kingdom

**Keywords:** neutron macromolecular crystallography, Monte Carlo simulations, neutron diffraction

## Abstract

Monte Carlo neutron ray-tracing simulations of time-of-flight (TOF)–Laue neutron macromolecular crystal diffraction (*n*-MX) using the *McStas* software package were performed for the upcoming NMX Macromolecular Diffracto­meter at the European Spallation Source. Splitting neutron rays that arrive at the crystal leads to dramatic improvements in event formation with minimal computational overhead. The simulated event-probability data were sampled using a new single-pass weighted reservoir-sampling method, and processed like real *n*-MX data using *DIALS*. The effects of air and beamstop scatter on simulated data were investigated.

## Abstract 2

Monte Carlo simulations of neutron protein diffraction experiments provide useful data that models neutrons interacting with instrument components, as well as the crystal diffraction itself. These data can be applied to instrument development, such as the commissioning of the NMX Macromolecular Diffractometer at ESS.

## 1. Introduction

The work detailed here answers the question: how can one perform a neutron experiment without neutrons? In the strictest, most mundane terms, it is impossible; but with a computer and a sophisticated enough understanding of the underlying physics, we can certainly model such an experiment with a precision that yields useful insight. Thus, the current standard is to make use of computational tools that are good at modelling experiments, thereby pushing the boundaries of those experiments. Such synergy is at the core of this work.

### 1.1. The NMX instrument at ESS

The European Spallation Source (ESS) is a European project to build what will become the world’s most powerful neutron spallation facility (Peggs, 2013 ▸; Garoby *et al.*, 2017 ▸). The NMX Macromolecular Diffractometer (Markó *et al.*, 2020 ▸) – the ESS’s flagship instrument for neutron macromolecular crystallography (*n*-MX) – is designed to benefit from a long neutron pulse and bright moderators, which are unique features of the ESS.

The neutron scattering factors of hydrogen and deuterium are on a par with those of carbon and oxygen, leading to accurate and refineable H-atom positions from *n*-MX data (Moody, 2020 ▸; Meilleur *et al.*, 2006 ▸). More commonly used methods such as X-ray MX, electron MX and cryo-electron microscopy do not provide precise H-atom positions (Ahmed *et al.*, 2007 ▸), which makes *n*-MX a truly complementary technique to these more widely available methods. However, *n*-MX remains experimentally challenging, so it has thus far been used on a limited number of macromolecules (Kono *et al.*, 2022 ▸). The ambition at ESS is to expand the utility of *n*-MX to a broader range of biologically relevant systems (Oksanen, 2026 ▸).

The ‘entry barrier’ to *n*-MX is having crystals that are not only large (on the order of 1 mm^3^; Budayova-Spano *et al.*, 2020 ▸), but also isotopically labelled, with hydrogen replaced with deuterium to eliminate the strong incoherent neutron scattering from ^1^H (although it is often sufficient to simply swap labile protons with deuterium in D_2_O). High neutron flux, as will be provided by the ESS, can greatly lower the barrier to entry by requiring smaller crystals. Since neutron sources have lower flux than X-ray sources, we want to take advantage of every neutron that is available. Thus, Laue diffraction techniques, which use a wider bandwidth of radiation, improve neutron economy (Niimura *et al.*, 1997 ▸), and time-of-flight Laue (TOF–Laue) techniques, which exploit the de Broglie wave nature of neutrons, are unique to neutron crystallography and can improve signal to noise and facilitate refinement (Bull *et al.*, 2014 ▸; Peters & Jauch, 2002 ▸).

NMX is also designed to overcome challenges in the *n*-MX experiment unrelated to the neutron source. Most *n*-MX instruments have detectors at fixed geometries in order to maximize angular coverage at the cost of limiting the resolvable unit-cell size (Schultz *et al.*, 2005 ▸; Keen *et al.*, 2006 ▸; Tanaka *et al.*, 2009 ▸). NMX, by contrast, will feature a variable-geometry detector setup to allow the resolution of reflections from crystals with large unit cells, *i.e.* by increasing the sample-to-detector distance. Trading solid-angle coverage per exposure to achieve this will nonetheless expand the *n*-MX technique to more systems.

High-quality simulation data allow us to test our ability to translate the promise of these innovations to real data well before the instrument opens. Fortunately, much of the groundwork has already been laid for simulating *n*-MX data.

### 1.2. Simulations for neutron instrument planning

There are a few software packages available for neutron simulations, including (but not limited to) *McStas*, *VITESS*, (Manoshin *et al.*, 2011 ▸) and *Geant*4 (Allison *et al.*, 2016 ▸). The former, *McStas*, is used in this work. *McStas* is an open-source software package for Monte Carlo (MC)-based ray tracing of slow neutrons and their interaction with matter (Lefmann & Nielsen, 1999 ▸; Willendrup & Lefmann, 2020 ▸, 2021 ▸). *McStas* has been used extensively for simulations of experiments at both current and planned neutron instruments (Udby *et al.*, 2011 ▸). It has been used to validate the design of every instrument at ESS so far, including NMX (Toft-Petersen *et al.*, 2025 ▸; Markó *et al.*, 2020 ▸).

A complete description of the underlying algorithms driving *McStas* is beyond the scope of this work and we recommend consulting the *McStas* documentation for a complete description (McStas Team, 2026 ▸). In *McStas*, a neutron is treated as a ray with an associated probability. The properties and trajectory of each ray are defined by three things: the source, which define the ray’s initial properties; the instrument, a list of all components that the ray can interact with, including the sample; and the monitors, which record the results. Each component in the instrument will have a calculated probability of interacting with a ray, modifying its momentum vector. If a ray crosses a monitor – in our case, an ideal 2D pixel detector – it is recorded in so-called ‘event mode’, which records the ray’s time of arrival (TOA)^1^, energy and total probability. However, we discard the recorded energy and calculate it from the TOA instead.

The computational logic behind *McStas* is simple but powerful. The instrument file containing the experimental parameters and components is converted into C code, which can be compiled by any C compiler such as *gcc* or *clang*. The simulations themselves are performed by simply running the resulting binary. McStas requires few external libraries, but parallelization – a necessity for this work – requires the MPI library. Because each simulation is that of an uncorrelated neutron ray trajectory, *McStas* is highly parallelizable and is well suited to high-performance computing (HPC).

### 1.3. Simulation of neutron MX experiments

As mentioned above, *McStas* has considerable utility in neutron instrument design, particularly for neutron optics (Toft-Petersen *et al.*, 2025 ▸; Herb *et al.*, 2022 ▸). It was also used in the optics design for NMX (Markó *et al.*, 2020 ▸). *McStas*can be used to perform realistic simulations of single-crystal diffraction experiments, but there are some important challenges that need to be addressed in order to properly simulate *n*-MX data.

(i) Sampling all of reciprocal space is difficult, as there are a large number of reflections that must be sampled, which can be over 10^6^.
(ii) Since MC simulations also model the probability of a protein crystal scattering a neutron ray, and such a probability is low, a large number of simulations are often necessary to fully cover reciprocal space.
(iii) As a result of the above two issues, computation – in terms of processing power and memory – can be costly.

For NMX specifically, an additional difficulty is performing simulations for an instrument under construction that has no real data to compare against (yet) and no comparable instrument exists elsewhere. The simulated data must nevertheless be realistic, and should be processed the same as any real *n*-MX data from a real instrument. This is also the benchmark that we will use.

A primary inspiration for simulating *n*-MX experiments is *MLFSOM* (Holton *et al.*, 2014 ▸), a program developed to generate X-ray MX data from a list of structure factors and a set of experimental parameters. The primary goals of *MLFSOM* were to better understand the sources of experimental error in MX experiments. Our current aims are different: to demonstrate the feasibility of using MC simulations for *n*-MX experiments and applying them to NMX instrument commissioning. Ultimately, however, our approach should also be applicable for modelling sources of experimental error in *n*-MX experiments.

## 2. Setup of computations with *McStas*

To perform *n*-MX simulations with *McStas*, one needs a complete description of the instrument to be modelled and a list of Bragg reflection data.

### 2.1. The instrument file

The *McStas* instrument file contains all of the component information for the instrument, from source to detector. Example instrument files used for NMX are given in the supporting information.

#### 2.1.1. Neutron source and moderator

*McStas* itself does not model neutron sources (for example fission reactors or spallation sources) or the interaction of sources with neutron moderators. These can be performed with other simulation programs such as *Geant*4 and *MCNP* (Kulesza *et al.*, 2024 ▸). Instead, it provides the distribution and geometry of the moderator output calculated elsewhere. Therefore, the neutron moderator, not the spallation target, is considered the ‘source’ of the neutrons for our simulations.

For the NMX instrument, we use the ESS ‘butterfly’ moderator component (Andersen *et al.*, 2020 ▸). One can specify the relevant beam port for this moderator in the instrument file; for NMX, this is beam port W1. The ESS has a long pulse length compared with other spallation sources (2.86 ms, compared with 0.2–0.5 ms at ISIS, J-PARC and SNS), leading to a significant uncertainty in the calculated TOF. For this reason, the sample position of NMX is located quite far from the source, at 157 m, to improve the TOF resolution (Markó *et al.*, 2020 ▸). TOA uncertainty is propagated in neutron ray-tracing simulations.

#### 2.1.2. Neutron guides

The NMX beam-delivery system is designed to deliver to the sample a maximum 5 × 5 mm beam of neutrons with ±0.2° divergence. The neutron guides are optimized for wavelengths over 1.8 Å and all of the optical components from the moderator to the experimental cave are modelled in *McStas*.

#### 2.1.3. Choppers

NMX uses two wavelength-defining disk choppers to provide a typical wavelength range of 1.8–3.55 or 2.2–3.95 Å (Markó *et al.*, 2020 ▸). In *McStas*, one can specify the energy range of neutrons coming out of the moderator to improve the calculation efficiency. In tandem, the phase of the choppers is calculated for the specified wavelength range. We can use this to simulate out-of-phase errors and deviations from ideal behaviour, including jitter effects.

#### 2.1.4. Collimation system

The collimating devices in the NMX cave, including slits and pinhole apertures, define the beam size and divergence at the sample. In addition, the effects of air scatter on the neutron beam must also be modelled from the last vacuum window as the last parts of the system are in air. We use the *Union* component system in *McStas* to define components of complex geometry (Bertelsen, 2017 ▸) and that are made of materials whose neutron interaction properties have been modelled using the NCrystal library (Cai & Kittelmann, 2020 ▸). Using the *Union* system enables multiple scattering between a complex set of component geometries. For example, the NMX pinhole is made of gadolinium and the full geometry of the pinhole system, along with the surrounding air, is defined as a *Union* component.

#### 2.1.5. Air scatter

The instrument components from the moderator to the beam-defining pinhole were modelled under vacuum. Scattering from air was included only up to the crystal in simulations using the ‘ideal’ beamstop. Simulations with the *Union* beamstop included air scatter around the entire volume of the instrument after the pinhole.

#### 2.1.6. Sample

The *Single_crystal* component in *McStas* as used in NMX simulations is described in greater detail in Section 2.2.1.

#### 2.1.7. Beamstop

For the initial simulations, a simple, idealistic beamstop concept was used, where any neutron ray that arrived at the crystal but did not scatter was discarded at that point. These simulations have very little background from air scattering and so are ‘unrealistic’, but nonetheless are useful for verifying the quality of the simulations up to the crystal. Later, more complex scenarios were simulated. First, we included a ‘perfect’ beamstop component – a disk of 1 cm diameter that absorbed any neutron that crossed it – set 1 cm downstream from the sample in order to probe the effects of air scattering after the crystal (not shown). Ultimately, a *Union* beamstop of 3.5 mm radius made of ^6^LiF powder in a 0.5 mm thick aluminium casing, set 1 cm downstream of the sample, was used as the prototype NMX beamstop. As the beamstop component and surrounding environment become more complex, the number of recorded events increases, as well as the computation time.

#### 2.1.8. Detectors

The NMX detector system (Pfeiffer *et al.*, 2016 ▸, 2026 ▸) is quite unique in that it consists of three fully mobile, wide-area triple-gas electron-multiplier (GEM) panels, in contrast to the fixed detector geometry of most other *n*-MX TOF–Laue instruments. The three detector panels are mounted on mobile robot arms with nearly complete coverage of the top hemisphere around the sample. To avoid collisions, the detector-positioning system of NMX uses predetermined detector configurations. The current setup has 11 configurations, but based on simulations and commissioning experiments these can be modified and other configurations added.

In *McStas*, each detector panel component is modelled as a 1280 × 1280 grid of pixels of size 400 × 400 µm. The detectors themselves were modelled as ideal monitors with 100% detection efficiency and no point-spread function. Simulations of neutron detectors can be performed in a limited manner in *McStas*, which we will explore in the future; more sophisticated GEM detector simulations have been described elsewhere (Pfeiffer *et al.*, 2019 ▸). The detector-box components were not modelled for these simulations.

Neutron monitor components in *McStas* can be configured to ‘restore’ neutron rays that hit them, effectively making the monitor transparent. All 33 planned detector positions can thus be simulated from a single instrument file when the restore_neutron option is turned on for each monitor component. Strictly speaking, this is not the same as simulating the 11 detector configurations independently, due to the fact that a neutron ray only interacts with the first monitor exit volume it crosses and ceases to interact with air after crossing it (but will still be measured by any other monitor it crosses). Nonetheless, the differences between simulations run with one configuration at a time and simulations with all configurations run simultaneously are insignificant, and simulations run with all configurations are ten times more efficient in CPU time.

### 2.2. Simulating protein crystal diffraction

The simulated protein crystal is defined by two parts: the *Single_crystal* component in *McStas* and the list of Bragg reflections.

#### 2.2.1. The *Single_crystal* component

The *Single_crystal* component in *McStas* is defined by a list of Bragg reflections, unit-cell axes, crystal size, lattice-plane spacing uncertainty Δ*d*/*d* and mosaicity η. For our calculations, the lattice-plane uncertainty and mosaicity were kept low (Δ*d*/*d* = 0.0001, η = 15 arcmin), as they tend to be low for protein crystals at room temperature. Crystal volumes of 1 mm^3^ were used. The crystal orientation was set so that the (100), (010) and (001) planes were perpendicular to the *McStasX*, *Y* and *Z* axes, respectively. Solvent surrounding the crystal and quartz capillary crystal holders, both of which are present in real *n*-MX measurements, were not modelled for these simulations.

The scattering process for a *Single_crystal* component works as follows. A neutron ray of a particular energy and vector interacts with the component, and its probability of interaction is calculated. When the neutron ray reaches the crystal, it is SPLIT (see below) and reflections that fulfil the Bragg condition are taken from the complete list (Section 2.2.2). The list of reflections chosen is effectively a ‘shell’ with the same radius as the Ewald sphere, with a shell thickness of 3σ deviation. For more details, see Section 8.4 of the *McStas* component manual (McStas Team, 2026 ▸). Scattering events were limited to the zeroth- and first-order scattering only, corresponding to transmission through the crystal and a single scattering event from the crystal, respectively. Including second-order scattering or higher increases the computation time by an order of magnitude, and such events have negligible probabilities and so can be ignored (Supplementary Table S4).

One of the biggest sources of computational inefficiency in *McStas* is the scattering of neutron rays from the crystal. The probability of a neutron scattering coherently off a protein crystal is already quite low. To improve efficiency, the SPLIT instruction in *McStas* is used for the *Single_crystal* component. In brief, a neutron ray that reaches the crystal is ‘split’ (best thought of as copy/pasting the neutron ray) into a defined SPLIT number of neutron rays, all of whose probabilities of interaction with the crystal will be calculated, and will propagate until the end of the instrument. Since the absolute number of neutron rays simulated relative to the total number of scattered neutron rays calculated is irrelevant, we can set the SPLIT value to a high number to greatly improve the computation efficiency.

The impact of using SPLIT on the *Single_crystal* component is striking (Table 1 ▸). SPLIT leads to a proportionate increase in the number of recorded events, with minimal computational overhead. The speed-up of SPLIT comes from caching the list of reflections that are in the Ewald shell, the calculation of which is expensive with large reflection lists. The SPLIT rays, all of which have the same properties as the original ray and thus would have the same reflections in their Ewald shells, then use the cached list, which is much faster. In addition, using SPLIT avoids re-computation of the neutron ray trajectory from source to sample, further improving efficiency. A SPLIT value of 10 000 or more leads to effectively complete sampling of the detector surfaces, as indicated by the jump in min[Σ(*p*)] from 1000 to 10 000.

Incoherent scattering from the crystal can be significant, particularly if the ^1^H content is high. In this work, we are only considering perdeuterated crystals in D_2_O, and thus the total incoherent scattering is relatively low. Absorption, coherent scattering and incoherent scattering cross-sections of protein crystals are user-provided and included in the list of Bragg reflections (see below). They can be easily calculated from the molecular formula of the protein and knowledge of the neutron scattering and absorption cross-sections; the PeriodicTable Python library (https://github.com/python-periodictable/periodictable) nicely automates the process (see the supporting information). The contribution from D_2_O solvent inside the crystal is also included.

#### 2.2.2. List of Bragg reflections

*McStas* must be provided with a complete list of Bragg reflections, defined by a Miller index (*h*, *k*, *l*) and structure factor *F*^2^ in barns. Currently, *McStas* does not calculate crystal symmetry and assumes that the space group is *P*1. It also does not calculate Friedel pairs. When preparing a list of reflections for *McStas* calculations, it is thus necessary to provide a full sphere of reflections to a given resolution and not just the hemisphere one would obtain from taking a list of reflections with symmetry and expanding it to *P*1 in most programs. Any reflection not on the list will not be modelled.

There are a number of ways to calculate structure factors of protein crystals with neutrons. The open-source structural biology toolkit *Gemmi* (Wojdyr, 2022 ▸) is well suited for this, as is *phenix.fmodel* from the *Phenix* suite of MX software (Liebschner *et al.*, 2019 ▸). The latter tool has the advantage of modelling coherent solvent scattering automatically. Modelling solvent can also be performed with *Gemmi* in a few steps. However, both methods require manually adding Friedel pairs to the generated lists. Using *Gemmi* with *pandas* (Pandas Development Team, 2020 ▸) or *reciprocalspaceship* (Greisman *et al.*, 2021 ▸) can easily perform this operation.

Reflection lists for perdeuterated *Pyrococcus furiosus* rubredoxin (PDB entry 4ar4; Cuypers *et al.*, 2013 ▸) were used for this work. Experimental neutron data with refined D positions were used as a starting point. A complete list of neutron scattering factors to either 1.2 or 0.6 Å was calculated from the deposited coordinates and refined against the experimental structure-factor data to account for solvation. The Jupyter notebook used to generate the .lau reflection files with *Gemmi* is provided in the supporting information.

### 2.3. Hardware requirements

*McStas* output binaries use MPI for highly parallelized computations, and with the SPLIT instruction fewer simulations from source to sample are required. Simpler simulations, such as those without realistic beamstops and without large volumes of air, require fewer total ray-tracing simulations than those that do have these things. For the NMX instrument, a simple simulation of a rubredoxin crystal, with an ‘ideal’ beamstop that simply discards any neutron that does not scatter off the crystal, requires a minimum of ∼1 × 10^10^ simulations, and up to ∼5 × 10^11^ simulations for more complex cases (for example modelling the scattering from a realistic beamstop, see Section 5.2).

We make heavy use of HPC resources for these simulations, in particular using the LUMI supercomputing cluster in Kajaani, Finland. A simple simulation with *N*_simulations_ = 5 × 10^11^ and SPLIT = 11 000 takes approximately 30 min on the LUMI-C partition using 1280 CPUs, and processing time scales roughly linearly with the number of CPUs used. HPC resources are not necessary to perform these calculations; a higher end, consumer-grade system is sufficient with enough memory, and will just take hours to days instead of minutes. More complicated simulations require more memory. The major source of memory consumption for our computations is the reflection list for protein crystals. Each MPI process loads the entire reflection list into memory. Thus, for large or very high-resolution protein crystals, the amount of memory required becomes unreasonably high, and out-of-memory errors can occur. We are currently working on improving this in *McStas*, but it is currently only an issue when performing massively parallel computations on HPC resources. Storage can pose a practical issue, as the output files for these simulations can run into the hundreds of gigabytes to terabytes. However, the output of multiple *McStas* runs can be combined easily, so one can offload the results of smaller runs onto offline storage if needed, and combine them later during processing.

Parallelization for this work is entirely performed with MPI. We are currently working on improving GPU acceleration for parallel computations in *McStas*. Currently, *McStas* has limited GPU support for NVIDIA GPUs, but it is not optimized for SPLIT *Single_crystal* components.

## 3. Converting event probabilities into events

When *McStas* monitor components record in event mode, the event is saved as an array with the neutron’s TOA, monitor pixel location and the probability of the neutron trajectory. *McStas* only calculates probabilities of neutron events, not actual events. To convert the list of event probabilities *p* into a list of events *N*, we must sample the list of probabilities in full, treating each event as independent. The best solution to this is weighted reservoir sampling with replacement (Park *et al.*, 2007 ▸; Shekelyan *et al.*, 2023 ▸).

Therein lies a problem: in order to fully sample reciprocal space for protein crystals in our *n*-MX simulations, we must record billions of events, totalling hundreds of gigabytes, if not terabytes, of data.^2^ Weighted reservoir sampling exists in statistical analysis software packages such as *NumPy*, but those methods use multi-pass algorithms that require the full list of event probabilities to be loaded into memory first. This is impossible in our case.

Instead, we rely on an algorithm for single-pass weighted reservoir sampling (Meligrana & Fazzone, 2026 ▸). This algorithm generates an event list of size *N* – the reservoir – from a list of probabilities *p*, but only requires reading each element once; in other words, *p* need not be fully loaded into memory. This algorithm has been proven to generate equivalent reservoirs to multi-pass algorithms. The method also allows random sampling from streamed data; that is, it can update the reservoir in real time as elements in *p* arrive, providing access to simulating streamed instrument data.

Our *stream_sampling_mcstas* software uses the *StreamSampling.jl* library (Meligrana, 2026 ▸), which itself is based on the OnlineStats library (Day & Zhou, 2020 ▸) in the Julia language (Bezanson *et al.*, 2017 ▸), to read a list of *McStas* event probabilities and generate a list of events with pixel location and TOA, much like a real neutron detector would (Finke, 2026 ▸). In this way we can generate ‘real’ event data from the *McStas* event-probability data, in the same format (NeXus TOFRaw; Könnecke *et al.*, 2015 ▸) that will be output when NMX comes online.

Another method that can be used to convert probabilities into events is to simply multiply each event probability by a large integer constant, thus converting each probability measurement into integer detector ‘counts’. These counts can then be binned and histogrammed. Doing this effectively simulates collecting an infinitely long dataset, as the event list *N* should look like *p* as *N* → ∞. We have opted for weighted random sampling in this work, as we do not plan on collecting infinitely long datasets.

## 4. Data reduction and analysis

### 4.1. Binning and histogramming

Neutron TOF–Laue data must be binned and histogrammed by TOF in order to be processed. We have developed the *ESSNMX* software package to process event data not just from NMX, but also our simulated data. It is based on the *scipp* package (Heybrock *et al.*, 2020 ▸), which provides numeric arrays with labels and unit measurements. *ESSNMX* converts measured TOA to TOF and outputs the binned/histogrammed event data in the NeXus NXLaueTOF format (NeXus International Advisory Committee, 2024 ▸). This is the same format that NMX will use when it comes online. *ESSNMX* also computes pixel positions for each detector based on the NXtransformation information in the TOFRaw format; NXtransformation is calculated from the *McStas* geometry in *stream_sampling_mcstas*.

### 4.2. Spotfinding, indexing and integration

Versions 3.27.0 and later of the *DIALS* software package (Winter *et al.*, 2018 ▸) include support for spotfinding, indexing and integration of *n*-MX TOF–Laue data from various instruments, as well as the simulated data from this work. *DIALS* imports the binned/histogrammed event/TOF data from *ESSNMX*. Spotfinding, indexing and integration are performed on the binned images. The indexing step is particularly useful for instrument commissioning, as we can test the sensitivity of measured detector geometry on indexing. The NMX detectors will be fully mobile in three dimensions, so reproducible positioning and accurate measurement of detector positions will be critical parameters for the reliable indexing of NMX data. The NMX detector positioners are rated to submillimetre precision.

Integration is performed with the *dials.tof_integrate* routine now available in *DIALS*. A number of reflection-profiling algorithms are available, including simple summation (which performs no profiling), so-called ‘seed skewness’ integration (Peters, 2003 ▸), 1D line profiling (Tomoyori *et al.*, 2013 ▸; Yano *et al.*, 2016 ▸) and 3D surface profiling (Gutmann, 2017 ▸). The latter two methods tend to give the best *I*/σ(*I*) for the simulated reflections, at the cost of (slightly) higher computation time.

The *Mantid* software package (Arnold *et al.*, 2014 ▸) can also be used to bin/histogram and reduce the simulated *n*-MX data. *McStas* can output the *Mantid*-style instrument data in the XML format needed to load the sampled data into *Mantid*. Both software packages will give similar results.

## 5. Results and discussion

### 5.1. Modelling Bragg diffraction

**Figure 1:**
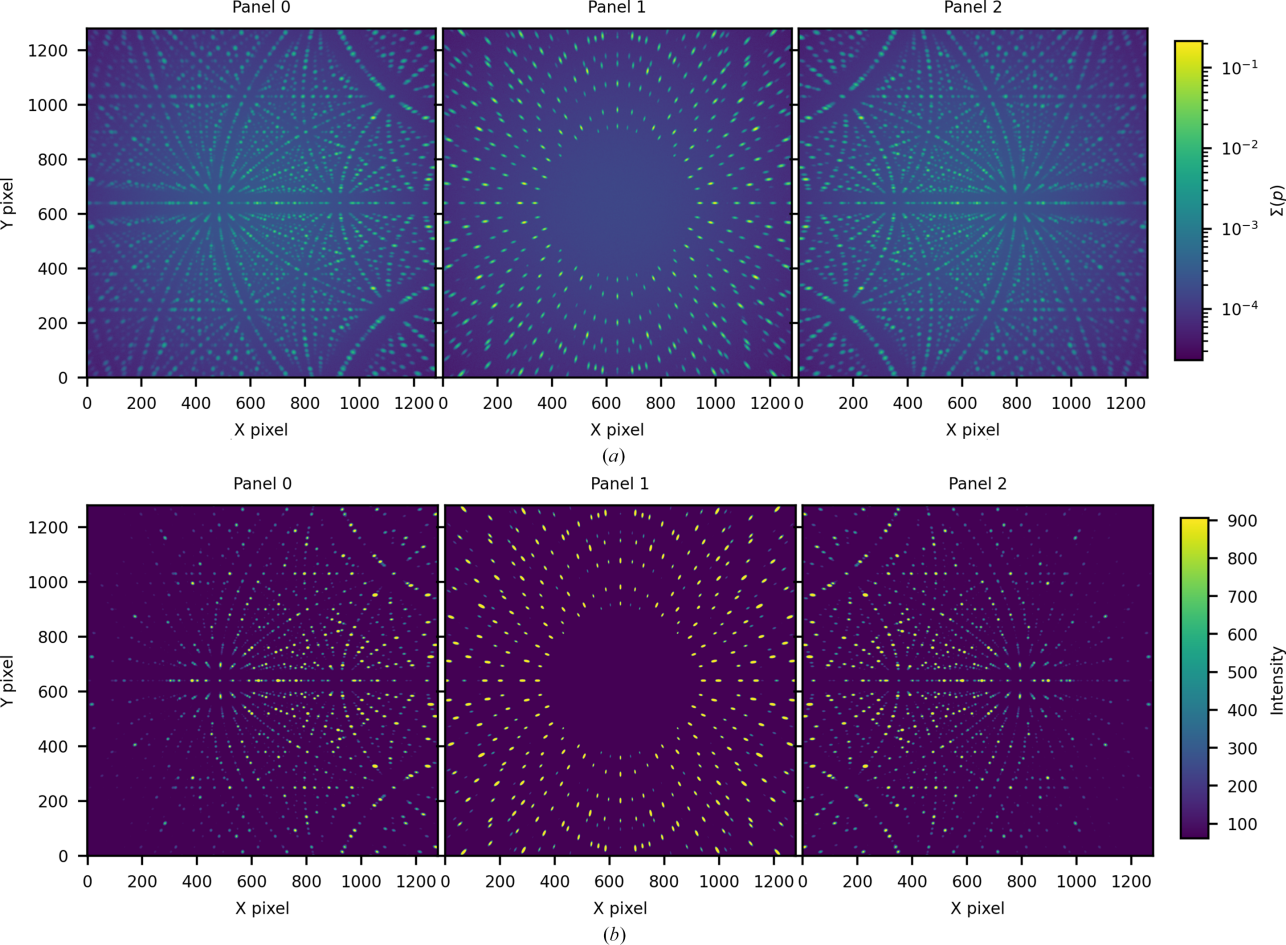
Rubredoxin diffraction simulation with ‘box’ detector geometry. *V*_crystal_ = 1 mm^3^, *N*_simulations_ = 5 × 10^11^, SPLIT = 11 000. (*a*) Sum of event probabilities by pixel. Note that the colour scale is logarithmic. (*b*) Sampled dataset from (*a*). *N*_sample_ = 1 × 10^8^.

Simple simulations of rubredoxin with NMX ‘box’ detector geometry were performed with an ideal beamstop (*i.e.* any neutron not scattered by the crystal was discarded at that point). Fig. 1 ▸ shows a histogram of the sum of event weight probabilities by pixel and the corresponding sampled dataset. As it is a Monte Carlo simulation, the number of simulated rays determines the completeness of the simulation space around the detectors. Table 2 ▸ shows the effect of *N*_simulations_ on the number of events recorded on the three detectors, *N*_events_. All other things being equal, a factor of ten increase in *N*_simulations_ leads to a tenfold increase in events measured on the detectors.

A visual depiction of the simulation results certainly looks, to the trained eye, like typical Laue diffraction for a protein crystal. When binned and histogrammed by TOF, each bin looks like monochromatic protein diffraction. A movie of the histogram binned by TOF shows the evolution of Bragg diffraction as a function of the incoming neutron ray wavelength (see the supporting information). These encouraging results were confirmed by processing the binned/histogrammed data in *DIALS*, showing that the simulated dataset is consistent with rubredoxin diffraction. The results are shown in Table 3 ▸.

The reservoir sample size *N*_sims_ has, unsurprisingly, the most significant effect in data-reduction results. Increasing the reservoir sample size is the statistical equivalent of collecting more data under otherwise identical conditions. A reservoir size of *N* = 5 × 10^7^–1× 10^8^ seems to be the point of diminishing returns. Importantly, sampling is perfomed across events recorded on all three detector panels, since the weighted probabilities all derive from the same source (scattering from the source), although *McStas* separates the results recorded on different monitors. As *N*_samples_ increases, *d*_min_ decreases in turn, in accordance with a larger *N*_samples_ corresponding to longer data collection, and weaker reflections take more time to collect. Laue images of simulations of varying sample size can be found in the supporting information. Assuming that the event list being sampled is randomly distributed (which it is in our case), one can see that the strongest reflections are almost fully sampled even at low *N*, much like what would be expected during data collection.

### 5.2. Simulating reality: other sources of scattering

The above, idealistic simulations of Bragg diffraction do not accurately reflect a realistic data collection. Even if the crystal is perfect, other sources of scattering from air, water, the beamstop, inaccurately placed components *etc.* contribute to the background of the measurement. Detector jitter, electronic noise, beam anisotropy, γ-rays from absorbed neutrons and cosmic rays are also sources of background in real *n*-MX measurements.

In *MLFSOM*, Holton and coworkers attempted to exhaustively model sources of measurement error in X-ray MX experiments using many semi-empirical relations (Holton *et al.*, 2014 ▸). *Ab initio* methods such as ours are less amenable to such exhaustive searches, as the computational complexity explodes quickly. This is not to say that sources of noise and background cannot be added after the fact. We will investigate this in the future.

**Figure 2:**
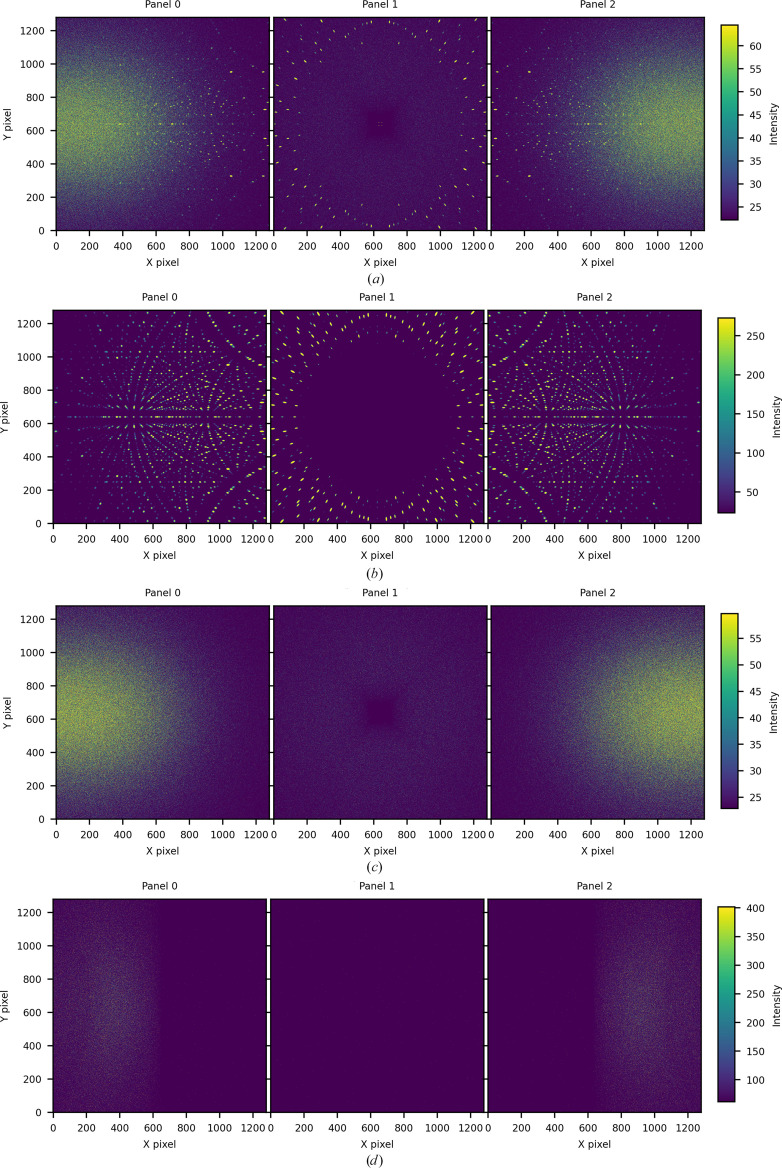
Rubredoxin simulation with ‘box’ detector geometry, with a 0.35 mm ^6^LiF beamstop in a thin aluminum tube (thickness 0.5 mm) placed 1 cm downstream of the sample. *V*_crystal_ = 1 mm^3^, *N*_simulations_ = 5 × 10^11^, SPLIT = 11 000, *N*_sampled_ = 1 × 10^8^. Sampling from four different conditional monitors: (*a*) monitoring all events, regardless of source; (*b*) monitoring scattering from the crystal; (*c*) monitoring scattering from air; (*d*) monitoring scattering from the beamstop.

Nonetheless, we can make some inroads into modelling some of the most significant sources of exogenous scattering. We can model a *Union* beamstop – one containing ^6^LiF in an aluminium tube, 1 cm downstream from the sample – and measure the effects of scattering up to the beamstop component. The results of this simulation are shown in Fig. 2 ▸. The effects of extra scattering from air and/or the beamstop are particularly prominent around *d* ≃ 1.5 Å, as indicated by the diffuse scattering cone there. Issues with sampling can also arise, as transmitted beam, or low-angle scattering, near the beamstop (centre of Panel 1) leads to extremely high neutron probabilities. A ‘mask’ around the beam centre, should a detector panel pass through it, or a higher sampling reservoir size, can overcome the high probabilities of the few affected pixels.

Modification of the SPLIT method for the *Union* beamstop simulation was necessary to avoid erroneous random events of high probability: on average 1000 times higher than other scattering events. Instead of setting a SPLIT on the *Single_crystal* component alone, the SPLIT value was divided between the *Union* master and the *Single_crystal* components, such that the product of the two SPLIT parameters was 11000 (*e.g.* setting SPLIT 11 for the *Union* component and 1000 for *Single_crystal*). This gave more reasonable results.

We can distinguish between events created from crystal scattering and elsewhere by using conditional monitors in *McStas* that only record events when certain conditions are met, for example a neutron scattering off the crystal or air or both (or neither). Fig. 2 ▸ shows the results of an NMX simulation with a rubredoxin crystal and a *Union* beamstop using conditional monitors: one from scattering off the crystal (Fig. 2 ▸*b*), air (Fig. 2 ▸*c*) and the beamstop (Fig. 2 ▸*d*). Air is the greatest contributor to exogenous scatter, while the contribution of the beamstop is relatively small. Conditional monitors will be invaluable when simulating more complex scenarios, such as the presence of H_2_O.

### 5.3. Simulating the detector geometries of NMX

So far, the simulations discussed here have been in a simple ‘box’ geometry, with the three detector panels huddled around the sample, about 35 cm away (see the supporting information). We have simulated rubredoxin diffraction with all 11 detector configurations, and processed the resulting TOF–Laue data with *DIALS*; the detailed results are given in the supporting information. It is possible to model all 33 detector positions at once due to the fact that a monitor in *McStas* simply records the neutrons that pass through it, and can do so unimpeded if desired. The ability to do this will prove very useful for data-collection strategy and planning. Notably, the effects of air scatter become significantly more severe for detector geometries where the panels are very far from the sample, as expected. Even with the same *N*_samples_, in configurations with detectors furthest away from the sample, such as configurations 9 and 10, exogenous scatter dominates the sampled data.

While, ostensibly, an interesting baseline check would be to look at rubredoxin diffraction data from neutron diffracto­meter instruments such as D19 at ILL (Cuypers *et al.*, 2013 ▸) or LADI at ILL (Weiss *et al.*, 2008 ▸) and see how they compare with the simulated data here using the same parameters (crystal size, beam divergence, pixel size *etc*.), such a comparison is outside the scope of this paper. This is because we would have to model the beam parameters and the entirety of those instruments, and each would be an independent study in itself. In addition, one of the more interesting points of comparison between neutron instruments is the various detectors used. We have decided not to model detector parameters for our current studies, aside from panel geometry and pixel size, until we have more concrete data from our own detector tests, which we are currently undertaking. Since our primary interest is in modelling the NMX diffractometer, we will compare our simulation data against experimental data at that time. Meanwhile, these are a useful guide for us as instrument providers and for our future users, and we hope that other *n*-MX instrument scientists can use our work as a starting point for simulating their own experiments.

## 6. Conclusions

We have described our initial work on simulations of the TOF–Laue *n*-MX experiment on the NMX Macromolecular Diffractometer at ESS using *McStas*. With the new single-pass weighted random sampling with reservoir, we can convert the *McStas* event-probability output into realistic data, as indicated by processing these data with *DIALS*. We will use these methods as a template for instrument commissioning, as well as for data-collection strategies and analysis.

## Supplementary Material

Supplmementary Figures and Tables. DOI: 10.1107/S205979832600584X/he5696sup1.pdf

Jupyter notebook explaining how to generate the reflection file for McStas from PDB data. DOI: 10.1107/S205979832600584X/he5696sup2.html

NMX instrument file for McStas: box configuration. DOI: 10.1107/S205979832600584X/he5696sup3.txt

NMX instrument file for McStas: all detector configurations. DOI: 10.1107/S205979832600584X/he5696sup4.txt

Supplementary movie S1. Evolution of Bragg diffraction as a function of the incoming neutron ray wavelength. DOI: 10.1107/S205979832600584X/he5696sup5.mp4

## Figures and Tables

**Table 1:** Effects of SPLIT on simulations of rubredoxin diffraction (*N*_sims_ = 1 × 10^11^)

SPLIT	Total No. of events recorded on detectors	Mean time per process† (min)	Min Σ(*p*) per pixel‡	Max Σ(*p*) per pixel
N/A§	6.85 × 10^4^	7.30	6.61 × 10^−21^	1.93
10	6.84 × 10^5^	7.78	2.90 × 10^−24^	0.50
100	6.84 × 10^6^	7.85	1.34 × 10^−24^	0.29
1000	6.86 × 10^7^	8.57	3.95 × 10^−25^	0.22
2000	1.37 × 10^8^	8.60	6.34 × 10^−22^	0.23
4000	2.74 × 10^8^	9.29	6.72 × 10^−19^	0.23
6000	4.13 × 10^8^	10.25	2.74 × 10^−12^	0.23
8000	5.52 × 10^8^	9.20	7.78 × 10^−09^	0.24
10000	6.86 × 10^8^	9.81	1.20 × 10^−07^	0.22

† Per MPI process; for these calculations, 1280 MPI processes were used.

‡ Nonzero probabilities.

§ No SPLIT.

**Table 2:** Effect of *N*_simulations_ on recorded *N*_events_

*N*_simulations_	*N*_events_	Time†
5 × 10^9^	3.81 × 10^7^	21 s
5 × 10^10^	3.79 × 10^8^	3.75 min
5 × 10^11^	3.77 × 10^9^	37.8 min
5 × 10^12^	3.77 × 10^10^	6.04 h
1 × 10^12^ (no SPLIT)	6.84 × 10^5^	1.25 h

† Using 1280 MPI processes.

**Table 3:** Results of *DIALS* processing on simulated datasets of rubredoxin diffraction with varying *N*_sims_ and *N*_samples_

								*I*/σ(*I*)	
Entry	*N*_sims_ †	*N*_samples_ ‡	No. of spots§	*N*_indexed_ ¶	Indexed¶ (%)	*d*_min_ (Å)	*N*_integrated_ ††	Summation††	1D profile††
1	5 × 10^9^	1 × 10^6^	279	269	96.4	2.07	190	35.88	39.59
2	5 × 10^9^	1 × 10^7^	2053	1842	89.7	1.56	1298	51.00	54.96
3	5 × 10^10^	1 × 10^6^	107	106	100.0	2.34	84	45.23	47.33
4	5 × 10^10^	1 × 10^7^	846	813	96.2	1.67	619	66.38	67.46
5	5 × 10^10^	5 × 10^7^	2298	2152	93.7	1.55	1533	90.03	88.35
6	5 × 10^10^	1 × 10^8^	6005	5643	94.0	1.20	2519	113.17	106.58
7	5 × 10^11^	1 × 10^6^	103	100	99.0	3.42	79	45.37	46.06
8	5 × 10^11^	1 × 10^7^	691	690	100.0	1.79	516	72.02	72.58
9	5 × 10^11^	5 × 10^7^	1809	938	51.9	1.62	623	107.1	105.8
10[Table-fn tfn10]	5 × 10^11^	5 × 10^7^	3740	3691	98.7	1.43	2429	108.54	109.97
11	5 × 10^11^	1 × 10^8^	2470	2397	97.0	1.51	1503	108.68	108.66
12†	5 × 10^11^	1 × 10^8^	1879	1629	86.7	1.67	1162	35.15	45.2
13	5 × 10^12^	1 × 10^6^	100	98	100.0	3.39	80	44.79	44.70
14	5 × 10^12^	1 × 10^7^	678	674	99.6	1.68	509	72.37	73.08
15	5 × 10^12^	5 × 10^7^	1734	1706	98.4	1.59	1196	109.02	108.41
16	5 × 10^12^	1 × 10^8^	2400	2355	98.1	1.51	1478	111.73	108.86

† Number of neutron simulations in *McStas*. All runs used SPLIT 11 000 at the *Single_crystal* component.

‡ Reservoir size for sampled dataset.

§ Number of spots found from *dials.find_spots* using spotfinding defaults, except for entry 10.

¶ From *dials.index*.

†† From *dials.tof_integrate*.

‡‡ Using more optimized spotfinding parameters in *dials.find_spots*. See Appendix *A*[App appa].

§§ With modelled beamstop.

## Data Availability

Primary *McStas* results from simulations can be accessed via SciCat through the DOI https://doi.org/10.17199/d9ca04b5-84e8-4428-ac75-ebf41066dfc7. A Jupyter notebook, calculate_structure_factors.ipynb, is available in the supporting information. This notebook demonstrates how to generate the list of structure factors in *Crystallo­graphica*.lau format from PDB entries. Two *McStas* instrument files for NMX, one with a simple ‘box’ geometry and ideal beamstop, and one with all detector configurations and a *Union* beamstop, are available in the supporting information. The *scipp*/*essnmx* software can be found at https://doi.org/10.5281/zenodo.10731810. The *StreamSampling.jl* software can be found at https://doi.org/10.5281/zenodo.12826684. The *stream_sampling_mcstas* software can be found at https://doi.org/10.5281/zenodo.19137556.

## Footnotes

1 We make a deliberate distinction between time of arrival (TOA) and time of flight (TOF). TOA is defined as the measured time between the last measured pulse and the time of event formation at the detector. By contrast, TOF is defined as the calculated time from neutron formation to the detector event, including correction factors. The primary distinction here is that the travel time for neutrons of relevant wavelength to arrive at the NMX detectors (about 72–144 ms) is longer than the ESS pulse rate (71.4 ms). In other words, TOA ≃ TOF mod *t*_pulse_.

2 Compressing large lists of floats of relatively uncorrelated data, as output by *McStas*, is inefficient.

## Appendix A Typical simulation workflow

(i) Generate the relevant .lau file for Bragg reflections, using the calculate_structure_factors.ipynb file. Save the .lau file in the same folder as the *McStas*NMX.instr file for the *McStas* simulation.

(ii) Run *mcrun*, an automated process that generates the *McStas* C code, runs the simulation binary and handles the output. This step is usually performed on HPC systems:The -c option forces recompilation of the binary. The --IDF instruction outputs the *Mantid*-style XML data for the source and monitor components. Setting a *McStas* variable declared in NMX.instr, such as XtalPhiY=0, on the command line ensures that all defaults are chosen without querying the user (necessary for batch processing). The output directory, set by -d, must not already exist or *mcrun* will exit. The NeXus output is saved in /path/to/output/mccode.h5.

(iii) Sample the *McStas* data:This will generate a NeXus file, sampling_output.h5, in TOFRaw format.

(iv) Bin and histogram the sampled data. Usually 50 bins are sufficient:

(v) Process the binned/histogrammed data with *DIALS*:We include the unit cell and symmetry for indexing, since they are known, but it is usually not necessary. In cases where there is little noise in the data, such as in the case of the ‘ideal’ beamstop, running *dials.find_spots* with default parameters sometimes gives erroneous results. Setting the following parameters can help:
