# Supplementary material for: Simulating neutron protein crystallography experiments: applications to the development of the NMX instrument at ESS
Source: Acta Crystallogr D Struct Biol. 2026 Jun 21;82(Pt 7):751–60. doi: 10.1107/S205979832600584X (PMC13317687; doi:10.1107/S205979832600584X)
Supplement: Supplementary file 1 [file d-82-00751-sup1.pdf]

Supporting Information for: Simulating Neutron Protein Crystallography Experiments: Applications to the Development of the NMX Instrument at ESS

M. Bertelsen, P.K. Willendrup, S. Yoo, A. Meligrana, D. McDonagh, J. Bergmann, E. Oksanen, A.D. Finke

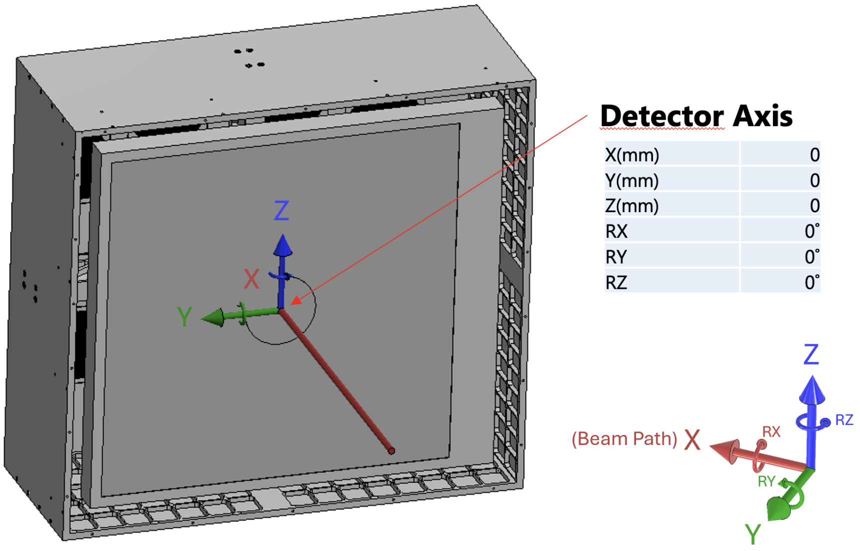

Figure S1: Coordinate system for detector panels. The X-axis is the neutron beam path. The Z-axis is parallel to gravity. The origin of the axis labels corresponds to the sample position. Measurements in Tables S1 and S2 are relative to the center of the detector panel. Rotations are according to the right-hand rule.

Table S1: Positions of Detectors for NMX Configurations (Box and Configurations 1–5)

Panel		Box	C1	C2	C3	C4	C5
Panel 1	X (mm)	250	270	331	336	581	451
	Y (mm)	0	0	0	0	0	0
	Z (mm)	0	0	160	0	0	0
	RX (°)	-90	0	0	135	0	0
	RY (°)	0	0	0	0	0	0
	RZ (°)	0	0	0	0	0	0
Panel 2	X (mm)	320	85.85	0	0	241.61	187.68
	Y (mm)	0	235.86	331	237.59	470.25	0
	Z (mm)	0	160	160	237.59	0	464.52
	RX (°)	0	180	180	180	180	0
	RY (°)	0	0	0	-45	0	-68
	RZ (°)	0	110	90	90	58	0
Panel 3	X (mm)	250	-113	0	0	241.61	-318.91
	Y (mm)	0	-386.49	-501	-237.59	-470.25	0
	Z (mm)	0	122.85	160	237.59	0	318.91
	RX (°)	90	0	0	180	0	0
	RY (°)	0	-17	0	-45	0	-135

Panel		Box	C1	C2	C3	C4	C5
	RZ (°)	0	-110	-90	-90	-58	0

Table S2: Positions of Detectors for NMX Configurations (Configurations 6–11)

Panel		C6	C7	C8	C9	C10	C11
Panel 1	X (mm)	435.63	1001	848.9	1001	1001	342.36
	Y (mm)	116.73	0	-530.45	0	0	0
	Z (mm)	0	0	0	0	0	940.63
	RX (°)	180	0	0	0	0	0
	RY (°)	0	0	0	0	0	-70
	RZ (°)	15	0	-32	0	0	0
Panel 2	X (mm)	319.57	799.43	848.9	-707.81	799.43	-309.33
	Y (mm)	-424.08	907.21	530.45	707.81	0	0
	Z (mm)	0	0	0	0	602.42	952.01
	RX (°)	0	0	0	0	0	0
	RY (°)	0	0	0	0	37	-108
	RZ (°)	-53	65	32	135	0	0
Panel 3	X (mm)	-240.5	-707.81	-121.99	423.04	259.08	-819.97
	Y (mm)	-416.56	-707.81	-993.54	-907.21	0	0
	Z (mm)	0	0	0	0	966.89	574.15
	RX (°)	0	0	0	0	0	0
	RY (°)	0	0	0	0	75	-145
	RZ (°)	-120	-135	-97	-65	0	0

The following Python code can be used to convert the above measurements (in ESS coordinate system) to McStas coordinates:

```
def coords_to_mcstas(x,y,z,rotx,roty,rotz):
    from scipy.spatial.transform import Rotation as R
    x2, y2, z2 = y, z, x  # xyz (ESS) = YZX (McStas)
    rotation = R.from_euler("xyz", [rotx, roty, rotz], degrees=True)
    ang = rotation.as_euler("YZX", degrees=True)
    return x2, y2, z2, ang[0], ang[1], ang[2]
```

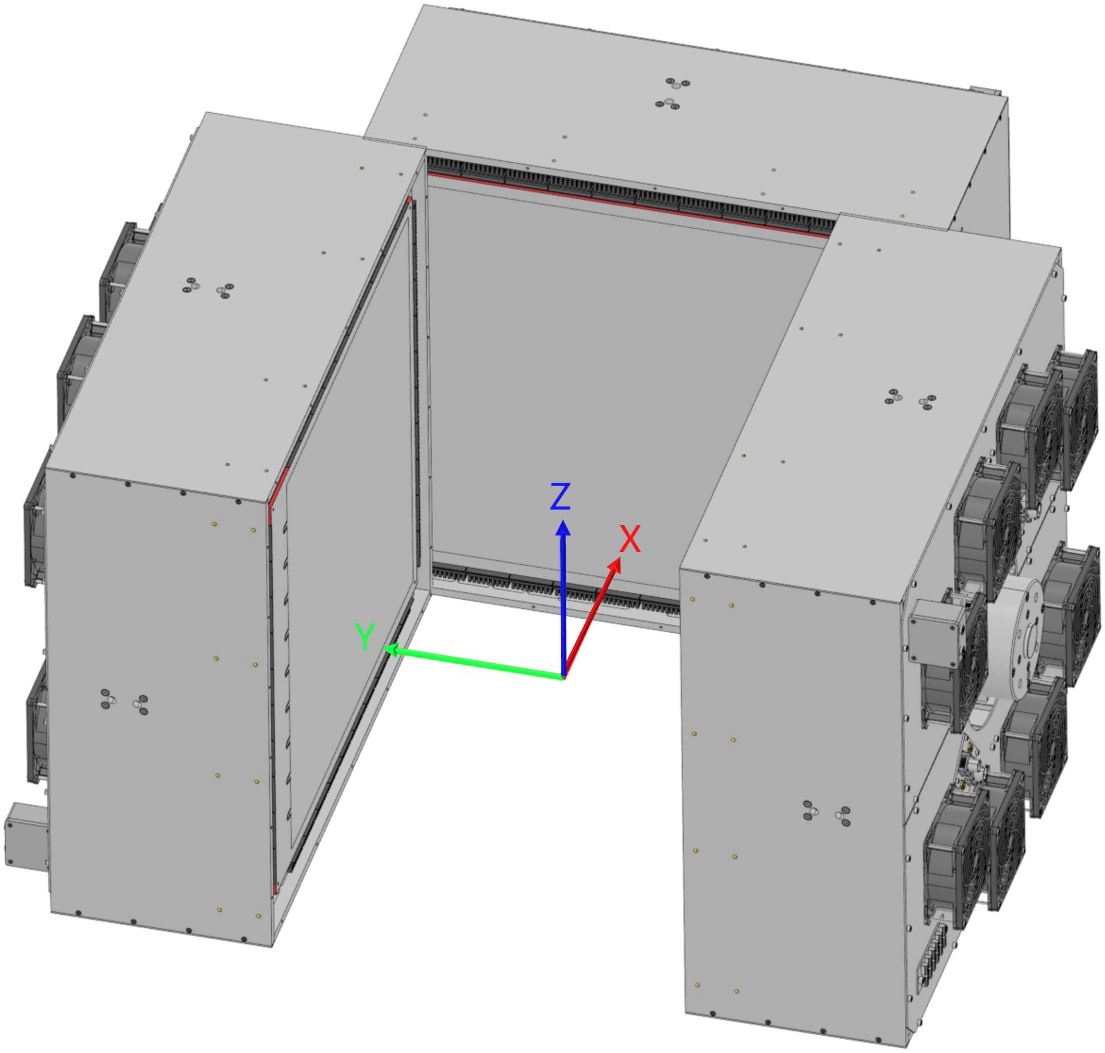

Figure S2: Detector "Box" Configuration.

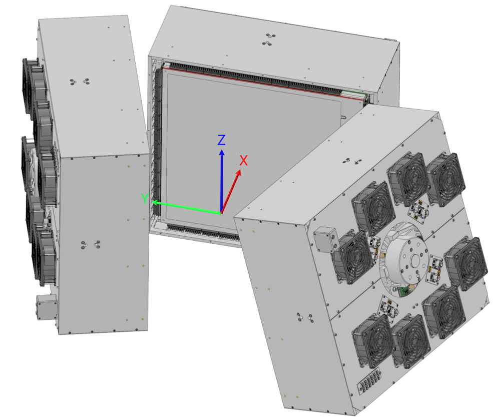

(a)

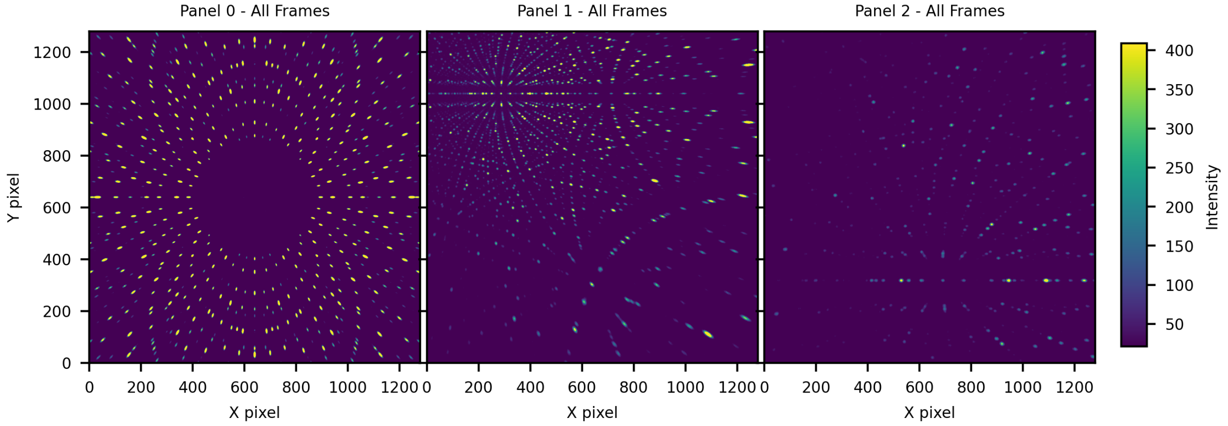

(b)

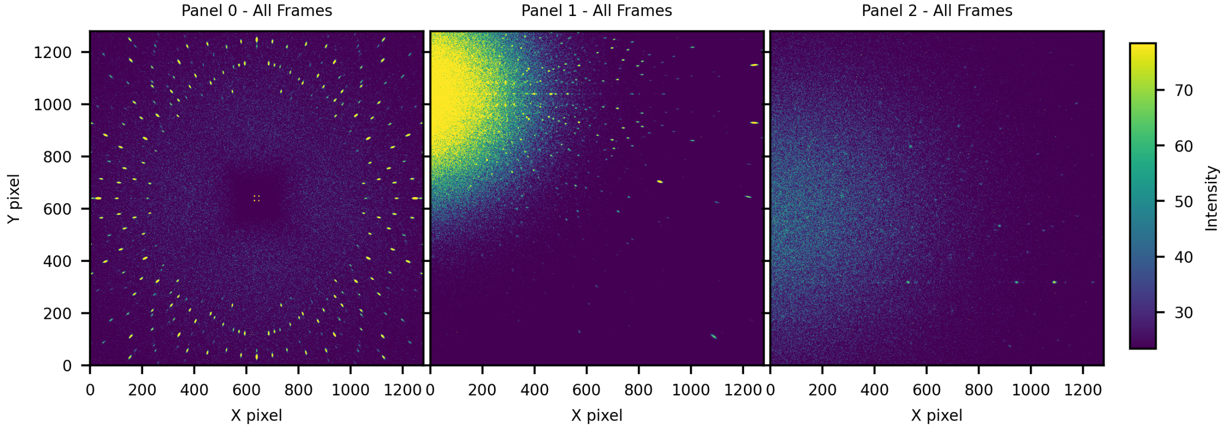

(c)

Figure S3: (a) Configuration 1. The X-axis is the beam path. The sample position is the origin of the axis labels. (b) Simulation of crystal only. (c) Simulation with beamstop. $N_{simulations} = 1e11$, $N_{samples} = 1e8$.

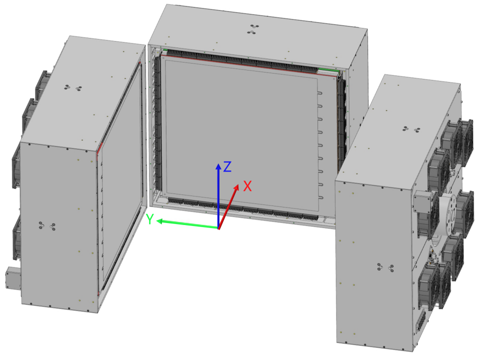

(a)

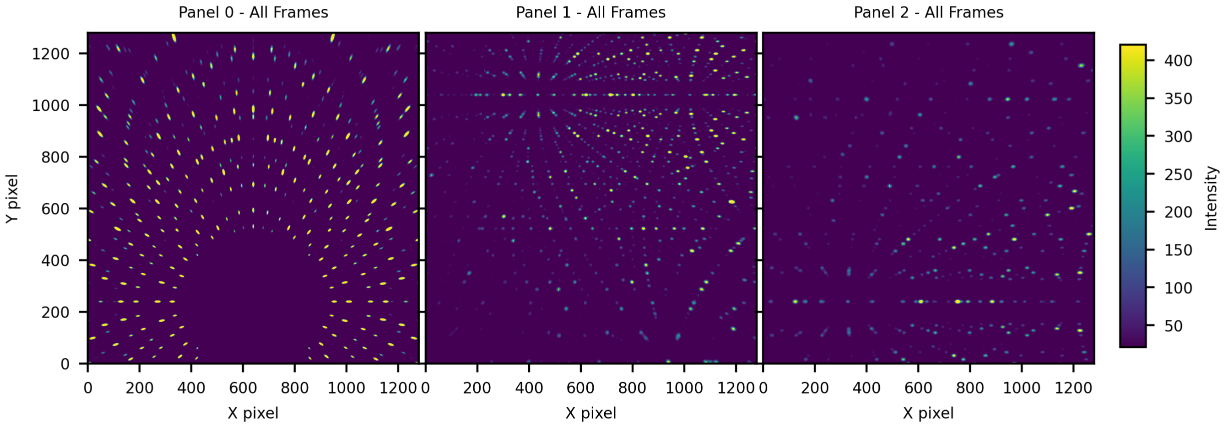

(b)

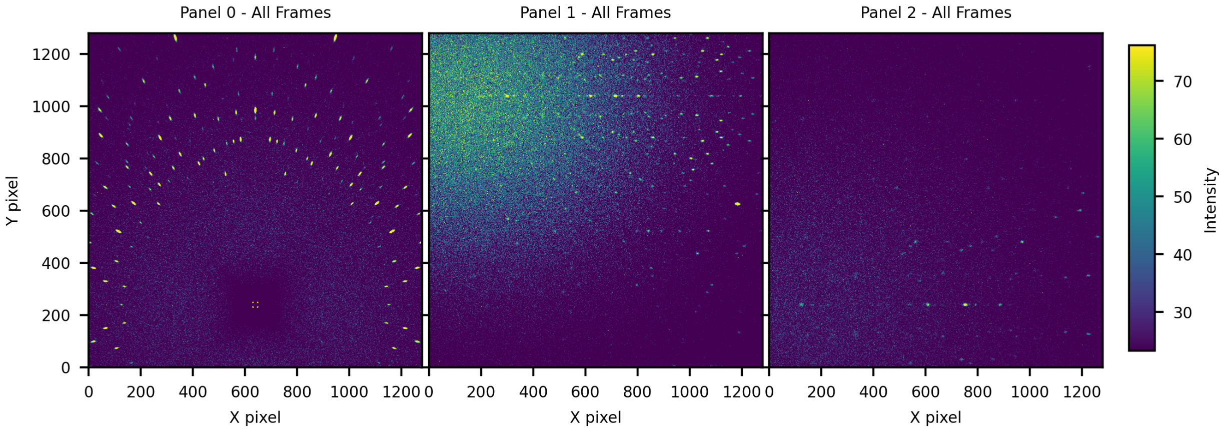

(c)

Figure S4: (a) Configuration 2. The X-axis is the beam path. The sample position is the origin of the axis labels. (b) Simulation of crystal only. (c) Simulation with beamstop. $N_{simulations} = 1e11$, $N_{samples} = 1e8$.

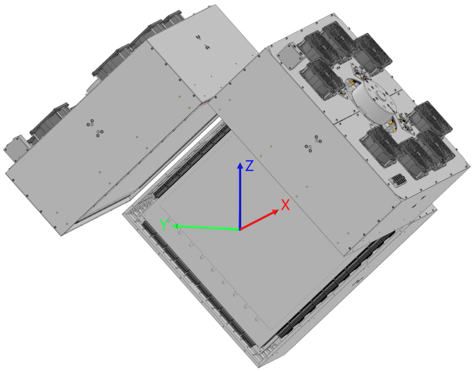

(a)

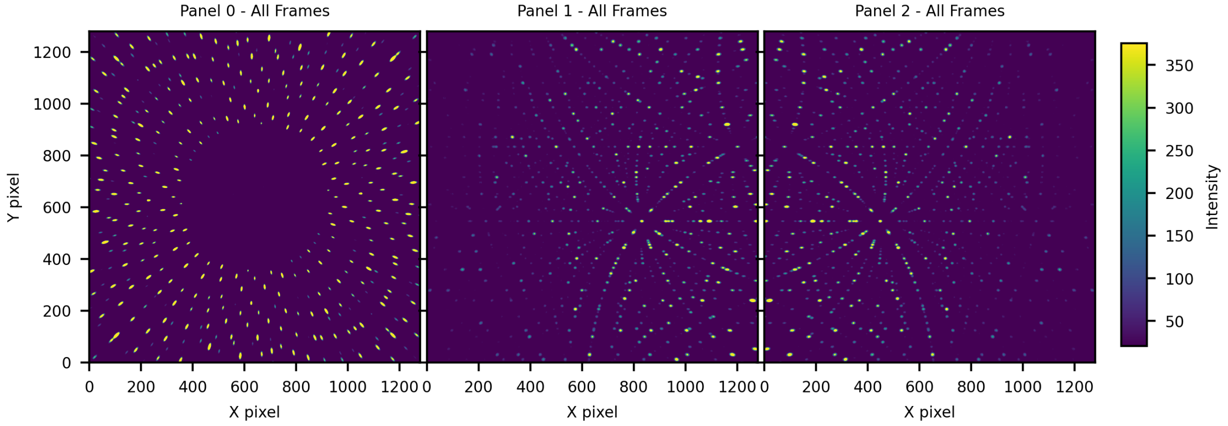

(b)

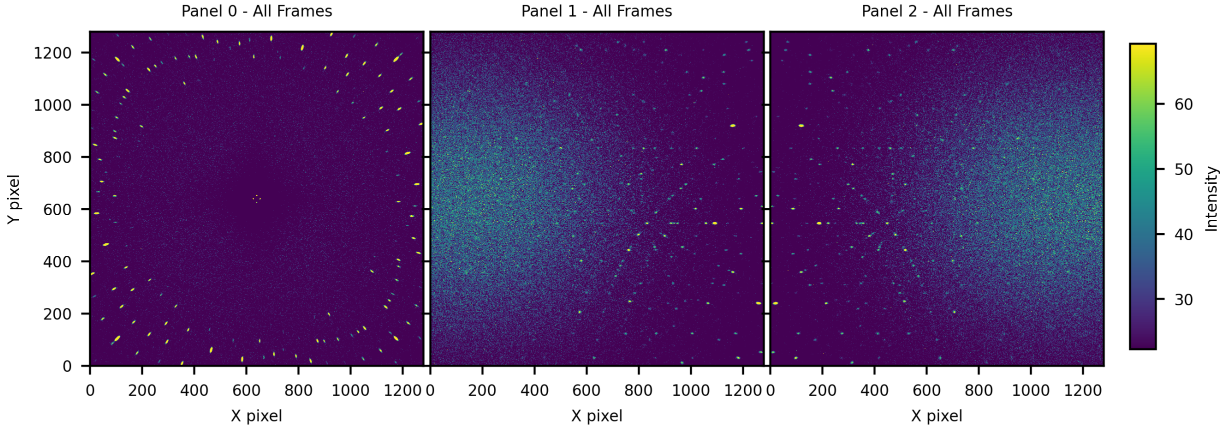

(c)

Figure S5: (a) Configuration 3. The X-axis is the beam path. The sample position is the origin of the axis labels. (b) Simulation of crystal only. (c) Simulation with beamstop. $N_{simulations} = 1e11$, $N_{samples} = 1e8$.

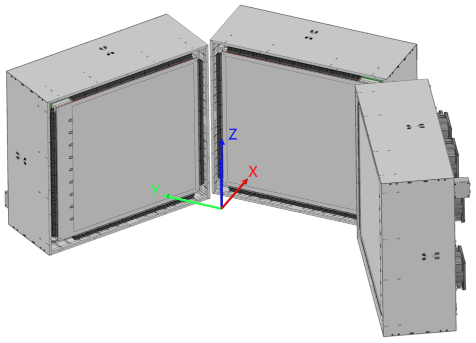

(a)

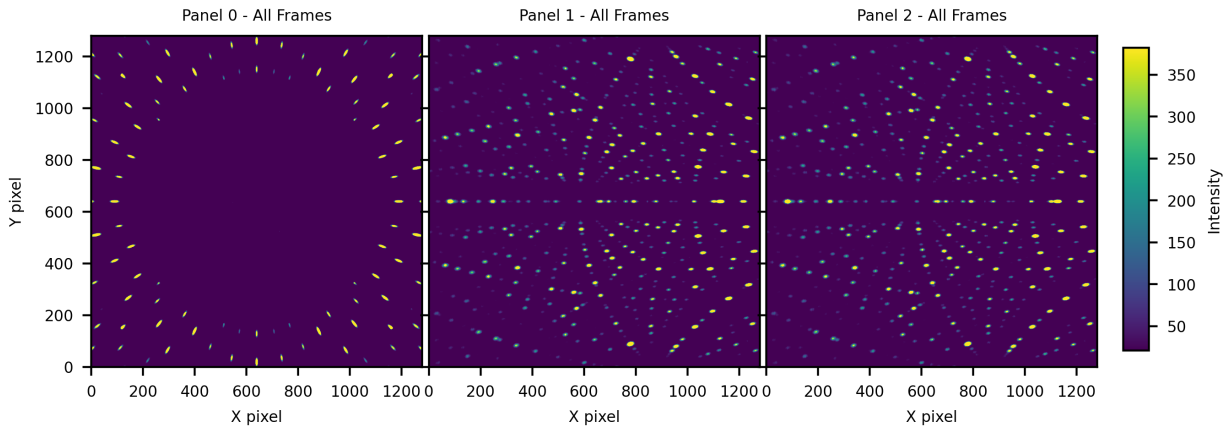

(b)

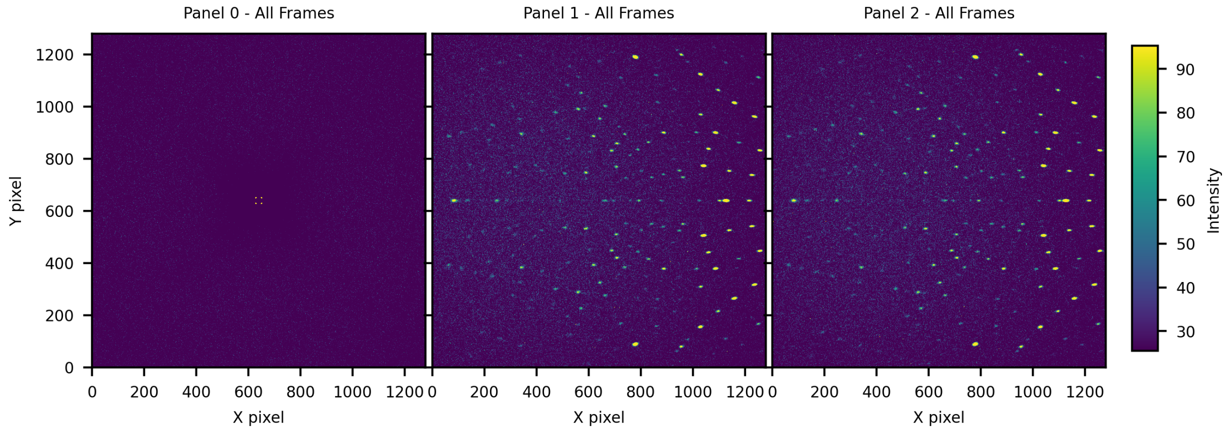

(c)

Figure S6: (a) Configuration 4. The X-axis is the beam path. The sample position is the origin of the axis labels. (b) Simulation of crystal only. (c) Simulation with beamstop. $N_{simulations} = 1e11$, $N_{samples} = 1e8$.

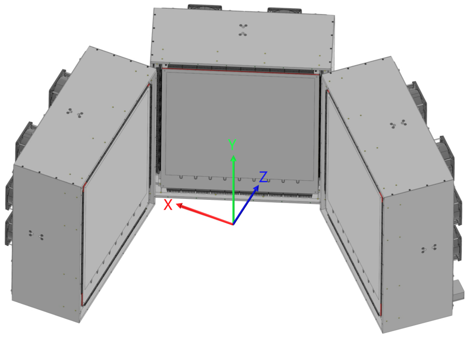

(a)

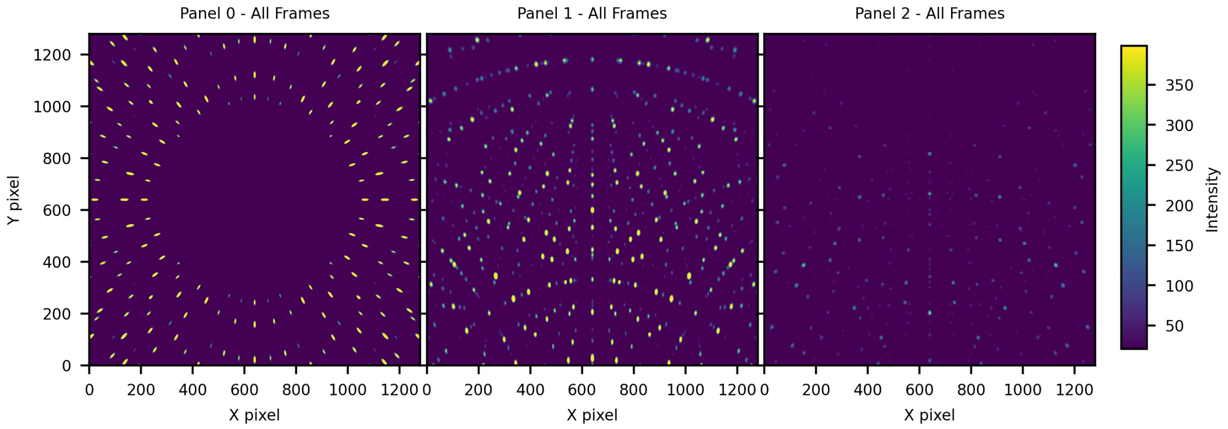

(b)

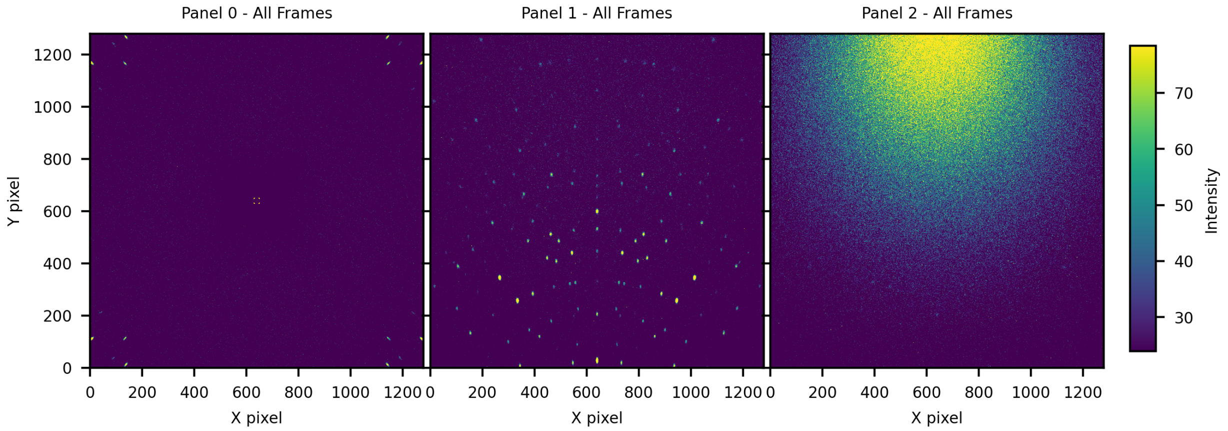

(c)

Figure S7: (a) Configuration 5. The X-axis is the beam path. The sample position is the origin of the axis labels. (b) Simulation of crystal only. (c) Simulation with beamstop. $N_{simulations} = 1e11$, $N_{samples} = 1e8$.

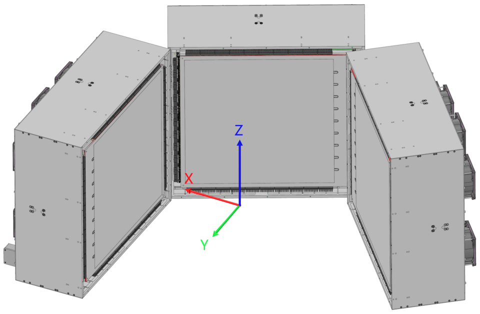

(a)

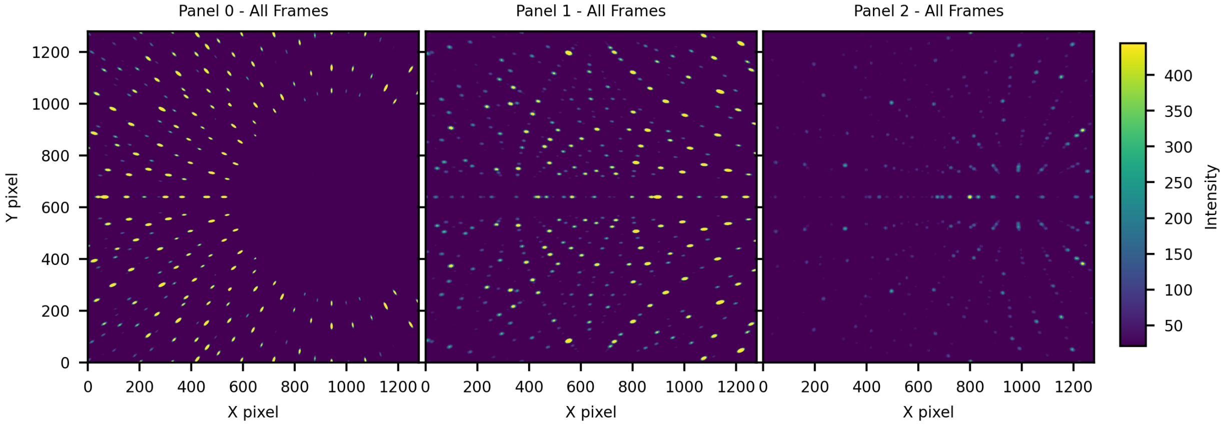

(b)

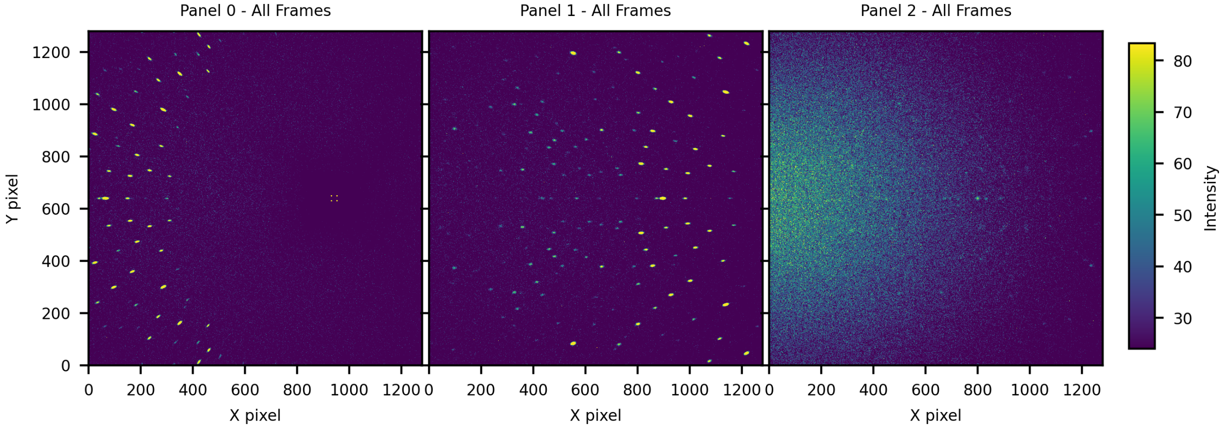

(c)

Figure S8: (a) Configuration 6. The X-axis is the beam path. The sample position is the origin of the axis labels. (b) Simulation of crystal only. (c) Simulation with beamstop. $N_{simulations} = 1e11$, $N_{samples} = 1e8$.

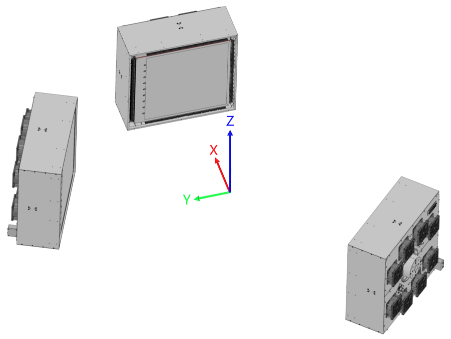

(a)

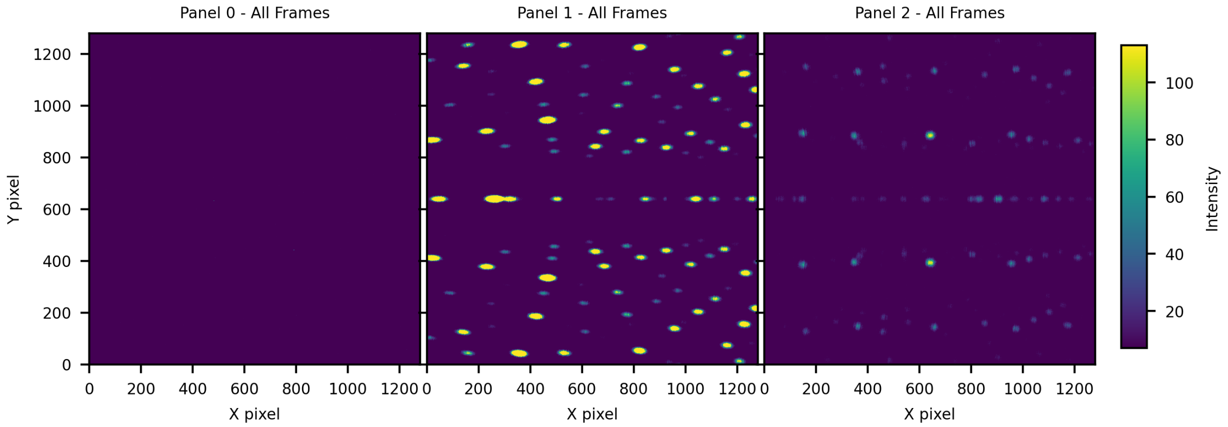

(b)

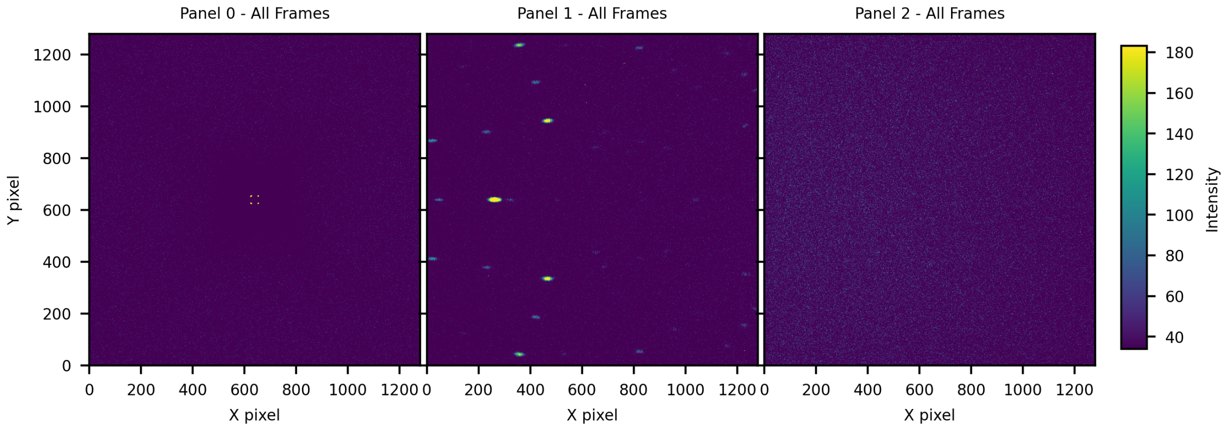

(c)

Figure S9: (a) Configuration 7. The X-axis is the beam path. The sample position is the origin of the axis labels. (b) Simulation of crystal only. (c) Simulation with beamstop. $N_{simulations} = 1e11$, $N_{samples} = 1e8$.

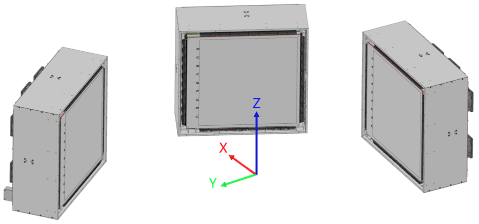

(a)

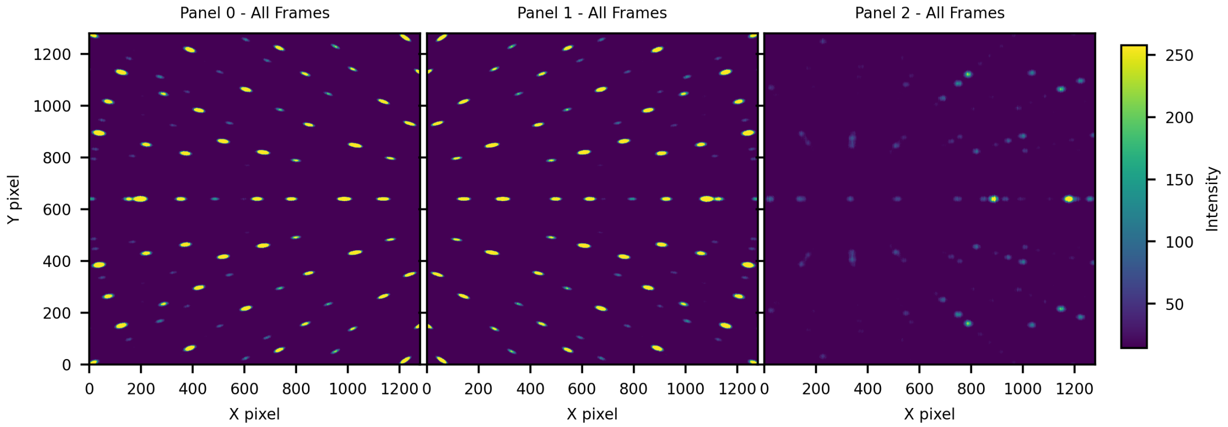

(b)

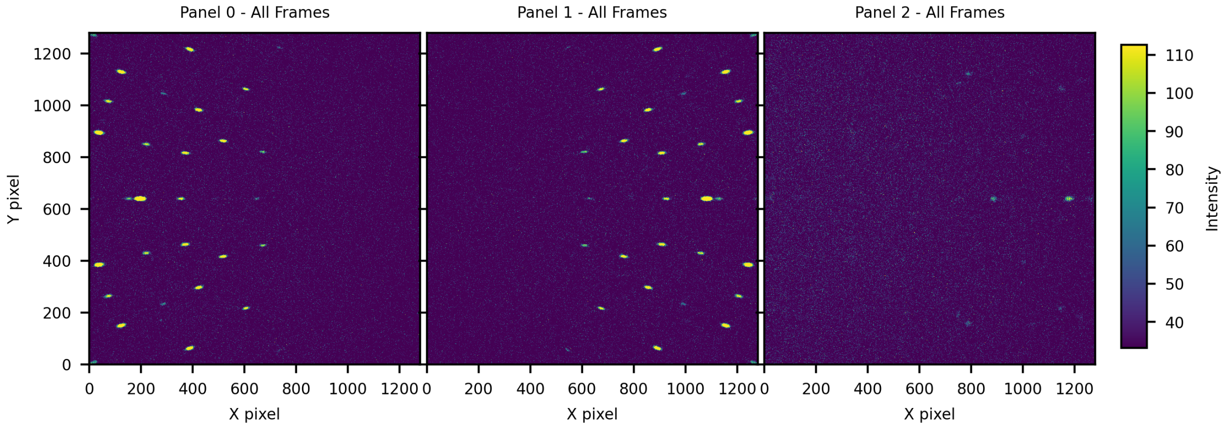

(c)

Figure S10: (a) Configuration 8. The X-axis is the beam path. The sample position is the origin of the axis labels. (b) Simulation of crystal only. (c) Simulation with beamstop. $N_{simulations} = 1e11$, $N_{samples} = 1e8$.

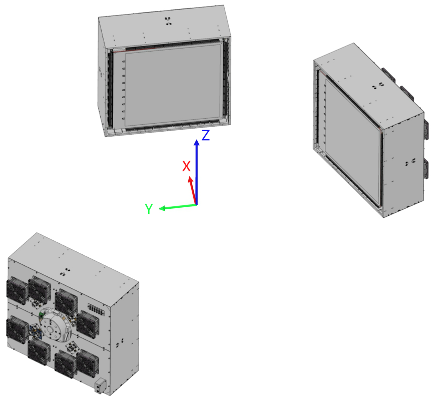

(a)

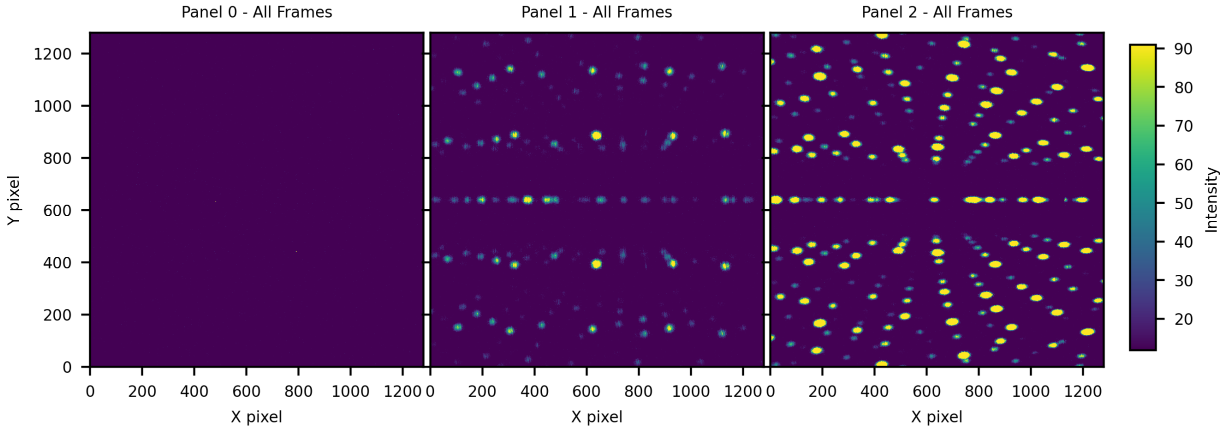

(b)

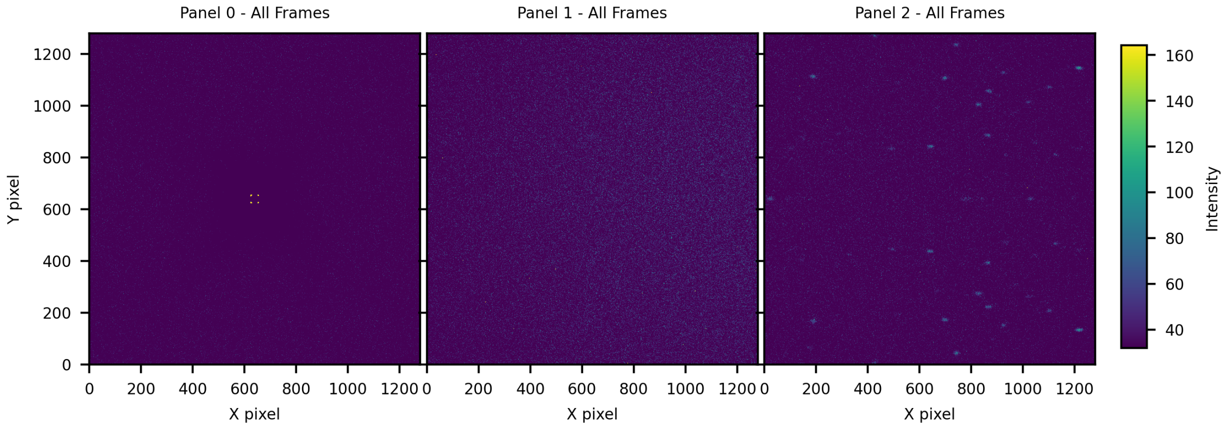

(c)

Figure S11: (a) Configuration 9. The X-axis is the beam path. The sample position is the origin of the axis labels. (b) Simulation of crystal only. (c) Simulation with beamstop. $N_{simulations} = 1e11$, $N_{samples} = 1e8$.

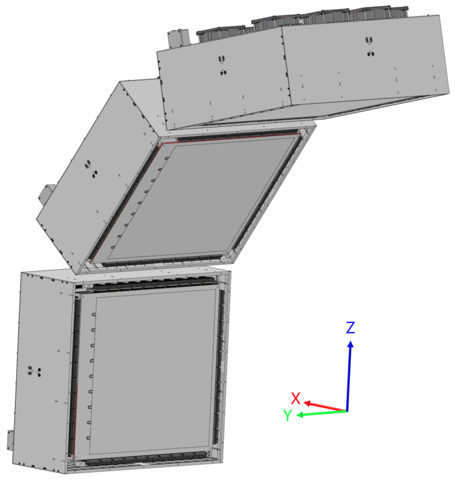

(a)

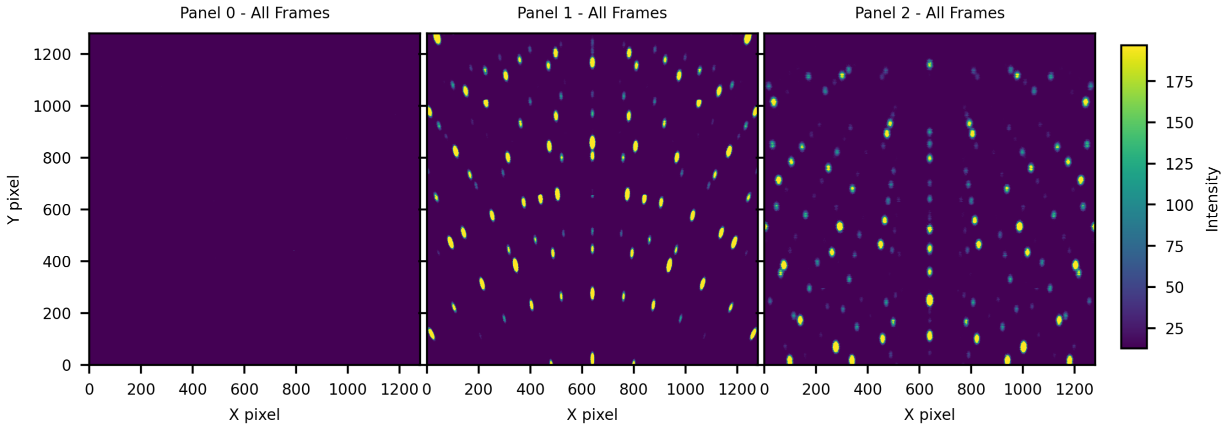

(b)

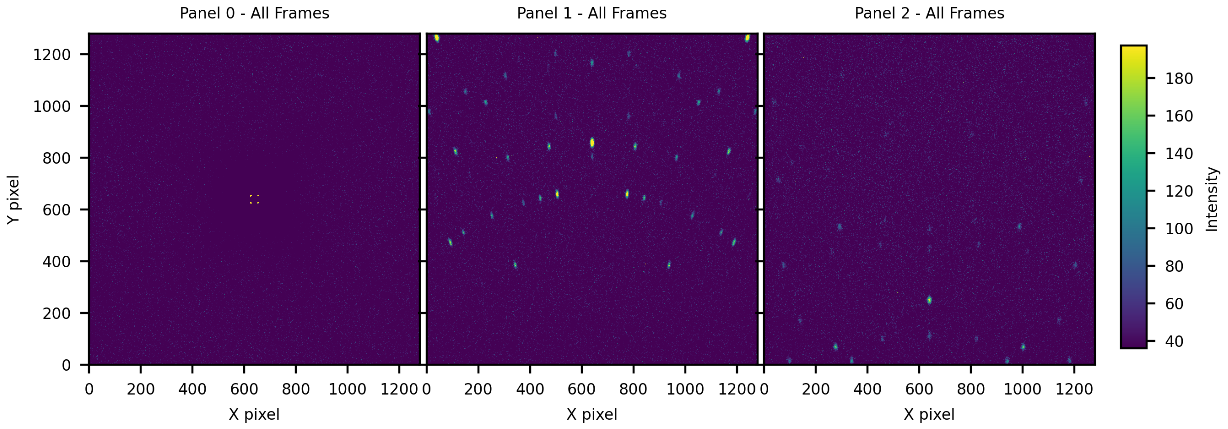

(c)

Figure S12: (a) Configuration 10. The X-axis is the beam path. The sample position is the origin of the axis labels. (b) Simulation of crystal only. (c) Simulation with beamstop. $N_{simulations} = 1e11$, $N_{samples} = 1e8$.

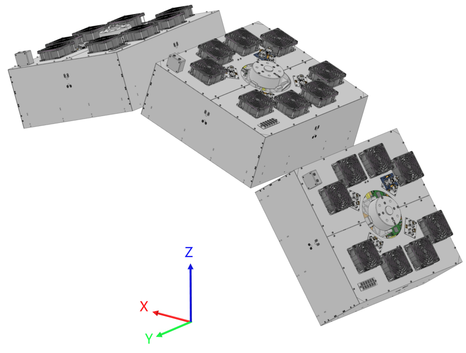

(a)

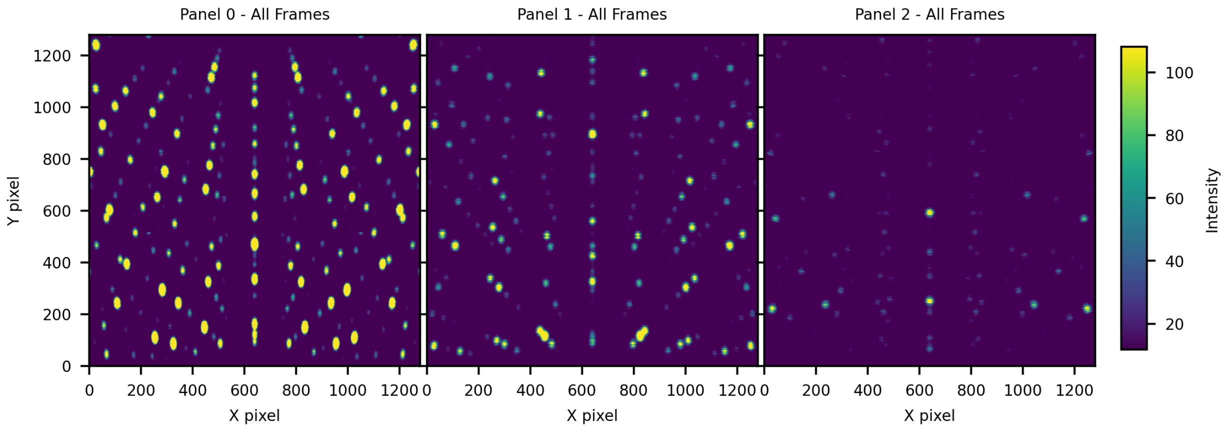

(b)

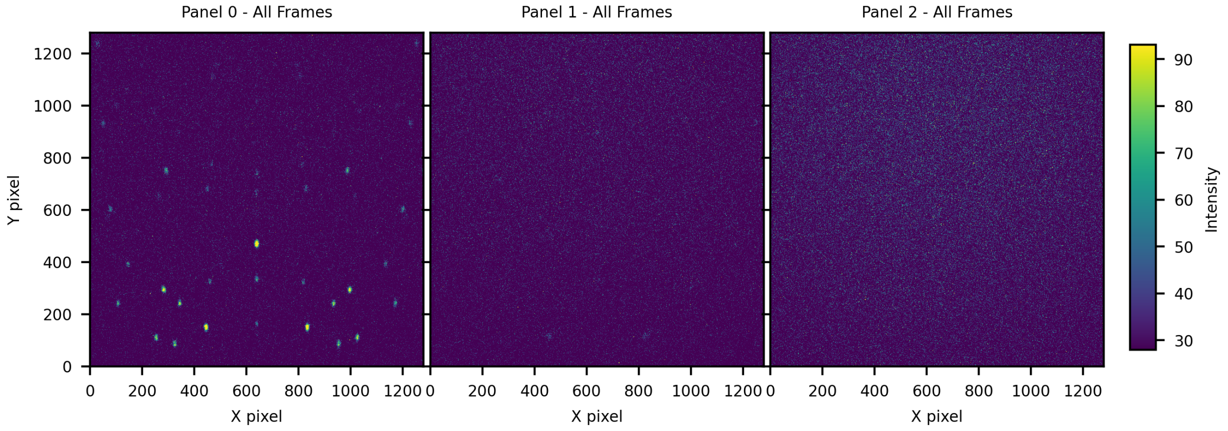

(c)

Figure S13: (a) Configuration 11. The X-axis is the beam path. The sample position is the origin of the axis labels. (b) Simulation of crystal only. (c) Simulation with beamstop. $N_{simulations} = 1e11$, $N_{samples} = 1e8$.

Table S3: Results of DIALS processing of Rubredoxin simulation of NMX detector configurations[a]

Entry	Configuration	Num spots[b]	$N_{indexed}$[c]	Indexed %[c]	dmin (Å)	$N_{int}/I/\sigma(I)$ summation[d]	N_{int}[d]$/I/\sigma(I)$ profile fitting[d]
1	1	2258	2077	92.0	1.42	1291/125.2	1280/144.9
2[e]	1	1241	1127	90.8	1.59	839/36.15	708/41.49
3	2	1531	1309	85.5	1.59	810/151.38	805/176.06
4[e]	2	801	724	90.4	1.67	592/38.17	549/42.44
5	3	1735	1593	91.9	1.55	1203/131.33	1196/153.39
6[e]	3	940	871	92.8	1.71	698/34.91	649/38.21
7	4	1414	1344	95.2	1.68	1032/159.16	1031/184.82
8[e]	4	629	530	84.4	1.94	490/51.31	475/56.22
9	5	990	728	73.6	1.44	413/141.82	406/167.83
10[e]	5	356	332	93.5	1.80	274/39.91	259/45.05
11	6	1035	736	71.2	1.56	421/147.87	414/175.86
12[e]	6	487	438	90.1	1.68	357/51.57	344/56.42
13	7	392	149	38.0	1.56	85/204.96	85/240.8
14[e]	7	102	69	68.3	2.35	49/95.3	48/110.96
15	8	468	415	89.1	1.59	249/238.03	247/276.62
16[e]	8	195	158	81.4	1.72	114/104.2	114/114.19
17	9	533	429	80.5	1.51	206/184.89	205/217.32
18[e]	9	144	90	62.5	1.96	64/65.71	62/75.13
19	10	323	305	94.7	1.58	152/23.85	65/50.65
20[e]	10	185	160	86.5	2.00	130/87.63	127/98.57
21	11	758	640	84.7	1.40	402/147.81	402/174.51
22[e]	11	148	145	98.6	1.68	106/60.44	103/70.54

[a] $N_{simulations} = 1e11$, SPLIT=11000, $N_{sampled} = 1e8$.

[b] Number of spots found from `dials.find_spots` using spotfinding defaults.

[c] From `dials.index`.

[d] From `dials.tof_integrate`.

[e] With modeled Union beamstop.

Figure S14: Histograms of probabilities for rubredoxin diffraction by pixel for the three detector panels. Note that the color scale is logarithmic. "box" detector geometry. $V_{crystal} = 1mm^3$. $N_{samples} = 1e11$. $SPLIT =$(a) 1, (b) 10, (c) 100, (d) 1000, (e) 10000.

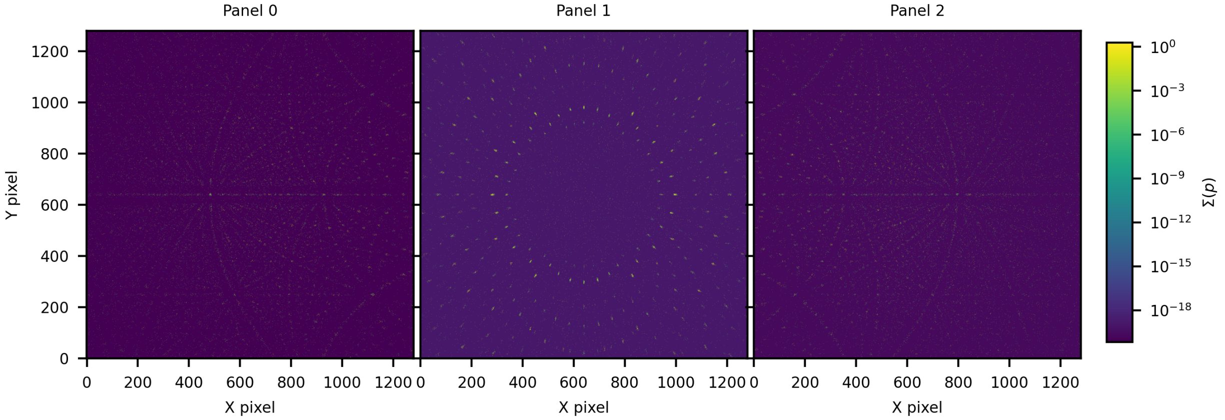

(a)

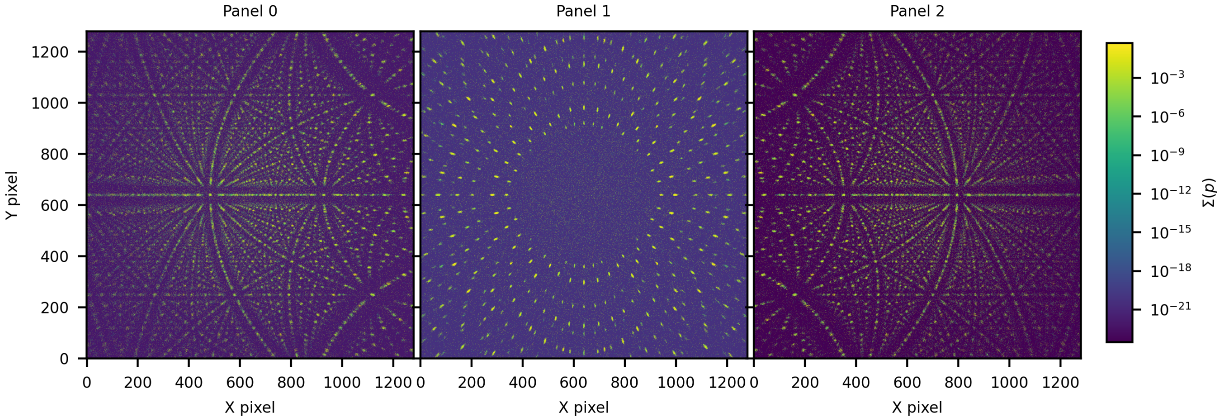

(b)

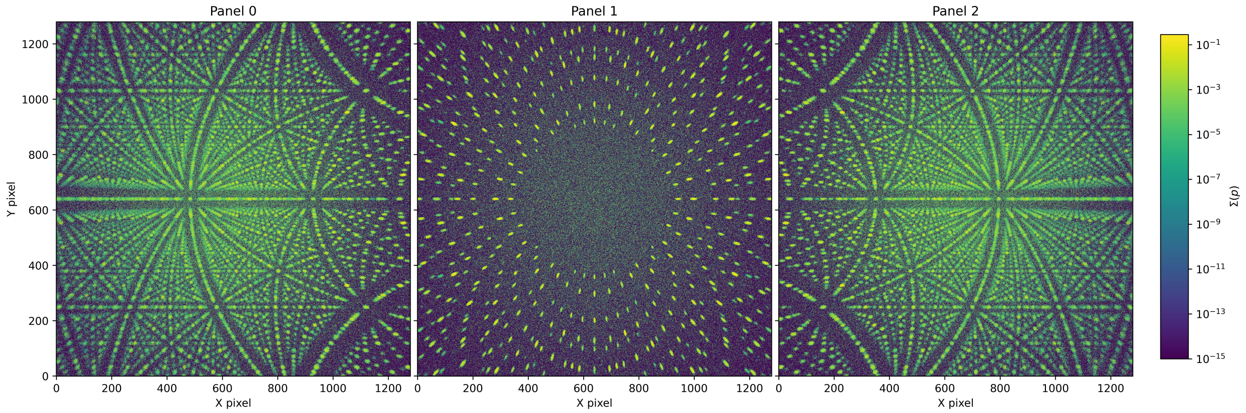

(c)

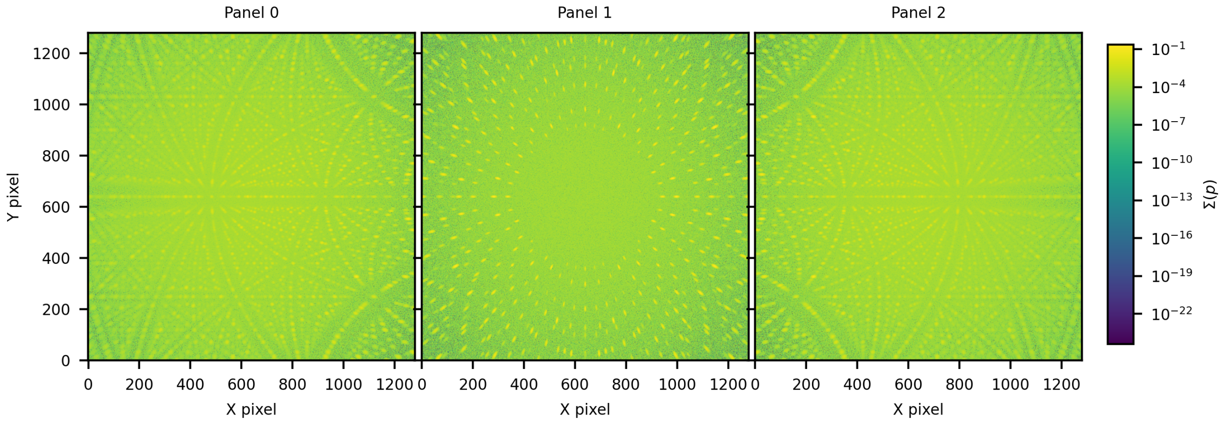

Panel 0
Panel 1
Panel 2
Y pixel
X pixel
Σ(p)

(d)

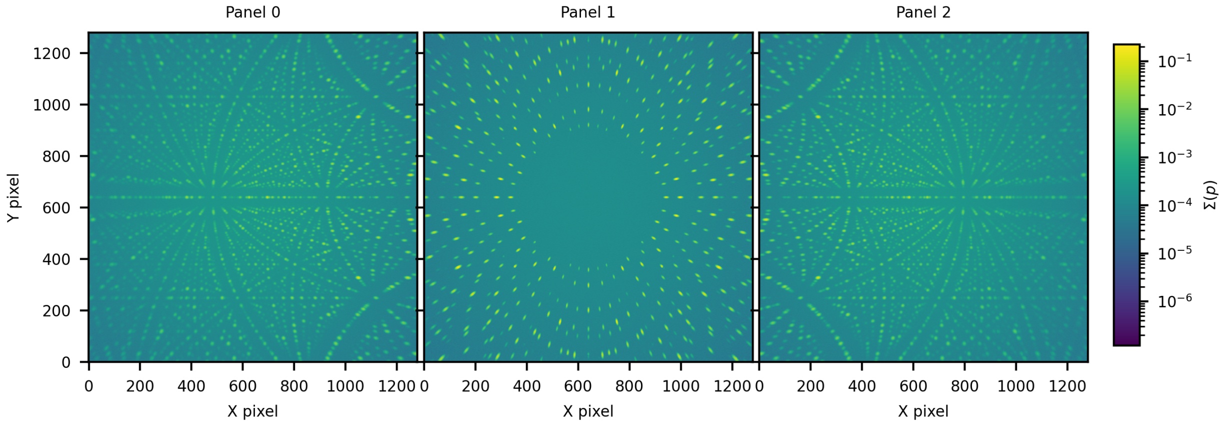

Panel 0
Panel 1
Panel 2
Y pixel
X pixel
Σ(p)

(e)

Table S4: Comparison of DIALS reduction statistics of enabling second-order scattering in the Single_crystal component in McStas[a]

	With second-order scattering [b]			First-order scattering only [c]		
Item	Overall	Low	High	Overall	Low	High
d_{min}	1.49	3.89	1.49	1.47	3.84	1.47
d_{max}	7.89	7.89	1.52	7.89	7.89	1.49
Reflns fully recorded	2391	107	7	2293	101	2
Reflns partially recorded	16	0	0	11	3	0
Reflns processed with summation	2407	107	7	2304	104	2
Reflns processed with profile fitting	2334	82	7	2234	74	2
Reflns failed in profile fitting	73	25	0	70	30	0
I_{bg}	2.33	8.71	1.65	2.39	9.15	1.29
$I/\sigma(I)$ (summation)	104.84	371.48	36.10	105.31	377.92	26.03
$I/\sigma(I)$ (profile fitting)	106.49	354.11	38.07	106.25	364.83	26.18
CC_{Pearson} (sum/prf)	0.99	0.99	0.81	0.99	0.99	1.00
CC_{Spearman} (sum/prf)	1.00	0.99	0.79	1.00	0.99	1.00

[a] Box detector configuration, ideal beamstop, SPLIT=11000, $N_{simulations} = 5e11$, $N_{sampled} = 1e8$.
[b] Computation time (12800 MPI processes): 54.1 min.
[c] Computation time (12800 MPI processes): 3.7 min.
